# Lineage-Associated Dichotomous Patterns in Codon Usage of Parasitic Cestodes

**DOI:** 10.3390/ijms27209282

**Published:** 2026-10-19

**Authors:** Seon-Hee Kim, Dongki Yang, Young-An Bae

**Affiliations:** 1 Research & Business Development Foundation, The State University of New York, Korea, Incheon 21985, Republic of Korea; sunnykim14@gmail.com; 2 Department of Physiology and Lee Gil Ya Cancer and Diabetes Institute, Gachon University College of Medicine, Incheon 21999, Republic of Korea; dkyang@gachon.ac.kr; 3 Department of Microbiology and Lee Gil Ya Cancer and Diabetes Institute, Gachon University College of Medicine, Incheon 21999, Republic of Korea

**Keywords:** codon usage bias, synonymous codon, tRNA repertoire, genome remodeling, translational adaptation, cestodes

## Abstract

Genome remodeling, accompanied by modifications in base composition and translational architecture, may systematically reshape codon usage patterns, contributing to adaptation to changing biological demands through altered translational efficiency. In the present study, codon usage and genomic tRNA profiles were comparatively investigated in species representing two cestode lineages with contrasting genome organizations and biological characteristics. Despite relatively well-preserved amino-acid frequencies, these cestode species exhibited clear inter-lineage divergence in synonymous codon usage. Diphyllobothriideans preferentially utilized C-ending synonymous codons, whereas cyclophyllideans favored the corresponding T-ending alternatives, forming distinct lineage-associated codon dialects. These divergent patterns were consistently retained across coding-sequence datasets ranging from genome-wide to highly curated orthologous sets. Nucleotide composition contributed substantially to codon usage, while multiple lines of evidence indicated additional selective and translational constraints. Genomic tRNA gene pools were greatly reduced in cyclophyllideans, particularly taeniids. However, relationships between synonymous codon preferences and corresponding tRNA gene counts varied among codon families, indicating that their evolutionary associations may not be uniform. Collectively, these findings suggest that distinct codon usage patterns have evolved in cestode lineages with contrasting genomic architectures, shaped by lineage-specific compositional environments and, potentially for certain codon families, translational factors. They provide a framework for investigating how such diversifications contribute to translational adaptation to nutritional and environmental changes.

## 1. Introduction

The standard genetic code consists of 64 nucleotide triplets (codons) that specify 20 proteinogenic amino acids and translational termination [1]. Most amino acids are encoded by two or more synonymous codons, collectively constituting a synonymous codon family [2]. Despite their equivalence at the amino-acid level, synonymous codons are rarely used at equal frequencies within genes or genomes, a widespread phenomenon generally referred to as codon usage bias (CUB) [3]. Furthermore, synonymous codons are not necessarily equivalent in their biological consequences, since they can differ in their interactions with the translational machinery, particularly according to the abundance and decoding properties of the tRNAs available for their translation [4,5]. Preferential use of codons efficiently decoded by available tRNAs can influence translational efficiency and accuracy, whereas cellular tRNA pools themselves may vary according to physiological and developmental states [4,5,6,7]. In this regard, synonymous codon choice can represent a biologically meaningful component of coding-sequence organization, potentially contributing to the efficiency and economy of gene expression through its effects on translational decoding.

CUB arises through the combined effects of multiple evolutionary and molecular processes. Mutational pressure and associated directional changes in genomic nucleotide composition provide major compositional constraints on synonymous codon choice at the relatively less constrained third codon positions, whereas natural selection can modify codon preferences according to gene function and translational requirements [3,8,9]. Gene length, expression level, local sequence context, recombination, and protein-related constraints may further contribute to variation in codon usage within and among genomes [9,10]. At a broader level, evolutionary changes in genome size and organization can alter the genomic environment within which synonymous codon usage evolves. Such genomic remodeling may involve changes in repetitive and non-coding DNA content, gene density and organization, nucleotide composition, and gene repertoires, potentially including multicopy tRNA genes [11,12,13]. Major changes in genome architecture are therefore likely to play an important role in reshaping both the compositional and translational environments influencing CUB, although their effects need not follow a universal or predetermined direction of codon preference.

Cyclophyllidea and Diphyllobothriidea encompass numerous cestode species of medical and veterinary importance and constitute evolutionarily distinct lineages characterized by contrasting morphological, ecological, and developmental traits [12,14,15]. Among these differences, their developmental and reproductive strategies are particularly notable, including differences in the capacity for asexual proliferation during larval development [12,14]. In several cyclophyllideans, particularly taeniids, asexual proliferation during larval development can generate large numbers of progeny, substantially enhancing reproductive potential within intermediate hosts [12,14,16]. Comparable proliferative development is uncommon among diphyllobothriideans, although a remarkable exception occurs in *Sparganum proliferum*, whose extensively branching plerocercoid larvae are capable of sustained somatic proliferation within host tissues [14,17]. These contrasting developmental and reproductive strategies plausibly impose different demands on cellular resource allocation and biosynthetic activity, potentially creating distinct selective environments for metabolic and translational efficiency.

These biological differences between the two major cestode lineages are accompanied by pronounced divergence in genome architecture. Available diphyllobothriidean genomes are generally several-fold larger than those of cyclophyllideans and contain substantially greater proportions of repetitive sequences, including transposable elements, whereas cyclophyllideans possess markedly condensed genomes with substantially reduced repetitive content [12,14]. Genes involved in multiple metabolic pathways were extensively reduced or lost during genome reduction, contributing to increased dependence on host-derived nutrients [12]. As previously investigated in bacteria [18,19], genome reduction may be accompanied by changes in nucleotide composition, potentially altering the genomic background within which synonymous codon preferences evolve, although comparable compositional remodeling has not been established in cestodes. More importantly, genome condensation may also reshape multicopy tRNA gene repertoires, as tRNA gene copy number and anticodon diversity have been shown to vary broadly with genome size across cellular organisms [13]. Such changes in tRNA gene abundance and anticodon composition could, in turn, modify the translational environment. Together, these contrasting biological and genomic evolutionary trajectories raise the possibility that synonymous coding organization and associated translational architecture have diverged between diphyllobothriidean and cyclophyllidean cestodes.

In the present study, the potential influence of genome condensation on synonymous codon usage was systematically investigated in representative diphyllobothriidean (*Spirometra erinaceieuropaei* and *Schistocephalus solidus*) and cyclophyllidean (*Echinococcus granulosus* and *Taenia solium*) cestodes. Using multiple complementary coding-sequence datasets to minimize potential biases associated with genome assembly, gene prediction, and dataset composition, synonymous codon usage patterns were compared between the two cestode lineages to assess the contributions of nucleotide composition and other evolutionary forces to their divergence. Genomic tRNA repertoires were also examined in the two lineages to gain insight into their relationships with synonymous codon preferences and the associated translational architecture.

## 2. Results

### 2.1. CDS Datasets

After removing sequences shorter than 300 bp, a total of 17,659 (recovery rate, 87.30%), 29,318 (74.12%), 9356 (93.70%), and 10,796 (87.45%) coding DNA sequences (CDSs) were obtained from the entire transcriptomes of *S. solidus* (20,228 entries), *S. erinaceieuropaei* (39,557 entries), *E. granulosus* (9985 entries), and *T. solium* (12,346 entries) available in WormBase ParaSite, respectively (genome-wide CDS [GW_CDS] dataset, Table 1). Based on pairwise similarity scores among decoded protein sequences, OrthoFinder identified 1325–1472 single-copy orthologs (SCOs; SCO_CDS dataset) from these GW_CDS datasets. CDS lengths in both the GW_CDS and SCO_CDS datasets differed significantly among donor species (Kruskal-Wallis test, *p* < 0.05), and those from *S. erinaceieuropaei* were markedly shorter than those from other cestodes (one-sided Mann-Whitney U tests with Holm correction, *p* < 0.001 for all comparisons; Table 1 and Supplementary Figure S1). Although *S. solidus* sequences also differed significantly from those of their orthologs, these differences were not consistently directional (two-sided tests, *p* < 0.001). Considering the number (22,162) and median length (1665 bp) of coding genes reported from a Japanese *S. erinaceieuropaei* isolate (BioProject PRJEB35375; scaffold N50 = 821 kb) [14], the much larger number (39,557) and smaller median size (426 bp [90–5841 bp]) of the current *S. erinaceieuropaei* CDSs from PRJEB1202, together with the lowest recovery rate among species, were likely attributable to incorrectly predicted gene architectures, mainly resulting from the highly fragmented genomic scaffolds (scaffold N50 = 4.6 kb). Meanwhile, the interspecific length heterogeneity was greatly reduced in the GenBank proteome-derived CDS (GP_CDS) dataset prepared using 43 individually curated *S. erinaceieuropaei* proteins from GenBank as queries (Kruskal-Wallis, *p* = 0.378; Table 1), further suggesting that many diphyllobothriidean CDSs retrieved from WormBase ParaSite are heavily truncated.

### 2.2. Base Compositions

Across all species, the coding DNA sequences in the GW_CDS dataset exhibited higher GC contents (49.67–53.37%) than those previously reported for the respective whole-genome sequences (42.1–46.0%) [20,21,22,23]. In the codon-based analysis, the values were highest at the first codon positions (GC_1_; 54.25–57.18%) and lowest at the second positions (GC_2_; 43.19–45.33%), whereas the third positions (GC_3_) showed values similar to those at the first positions (51.37–57.59%). The codon position-specific GC bias was repeatedly observed in the SCO_CDS and GP_CDS datasets, although the distribution ranges were progressively narrower in these selected CDSs than in the GW_CDS dataset (Table 1 and Supplementary Figure S2). The GC contents in these ortholog-based codon positions were broadly similar among cestode species despite differences observed at the whole-genome and genome-wide CDS levels. Nevertheless, it was apparent that *S. erinaceieuropaei* consistently exhibited significantly higher GC_3_ than other cestode species (one-way ANOVA and Kruskal-Wallis tests, all *p* < 0.001). Effect sizes indicated that species identity explained a non-trivial proportion of GC_3_ variance (η^2^ = 0.128, 0.052, and 0.099 for GW_CDS, SCO_CDS, and GP_CDS datasets, respectively), suggesting that the elevated GC_3_ values observed in *S. erinaceieuropaei* contributed substantially to the interspecific variance despite the broadly conserved GC compositions among cestode CDSs.

### 2.3. Lineage-Associated Divergence in Codon Usage Patterns

As summarized in Table 1, mean observed effective numbers of codons (ENC; ENC_obs_) ranged from 54.91 to 56.54 for the GW_CDS dataset, 55.68 to 56.92 for the SCO_CDS dataset, and 54.54 to 56.05 for the GP_CDS dataset. At the species level, *S. erinaceieuropaei* consistently exhibited the lowest values (indicating the highest bias) across the GW_CDS and SCO_CDS datasets, followed by *S. solidus*. In contrast, cyclophyllideans showed relatively higher ENC_obs_ values. These differences were statistically significant across all datasets (Kruskal-Wallis test, *p* < 0.001), with notable shifts between diphyllobothriideans and cyclophyllideans (Mann-Whitney U test, *p* < 0.001). However, the associated effect sizes remained small to moderate (rank-biserial correlation *r* = 0.21–0.35), suggesting that average bias alone is not the primary differentiator between these two groups. Instead, the quadratic relationship between ENC_obs_ and GC content at the third positions of synonymous codons (GC_3S_), modeled with a second-order polynomial, revealed significant differences among species and between pooled lineage groups. In the GW_CDS dataset, the ENC_obs_-GC_3S_ relationship was highly significant at both the species (*F*(6, 67,117) = 116.98, *p* < 0.001) and lineage (*F*(2, 67,123) = 70.11, *p* < 0.001) levels, explaining a non-trivial portion of the variance ($\eta_{p}^{2}$ = 0.12 for species). While this interaction persisted in the SCO_CDS dataset (*p* < 0.001), it was completely attenuated in the GP_CDS dataset (*p* = 0.932). This suggests that additional functional constraints may contribute to more similar ENC_obs_-GC_3S_ relationships among the sampled cestodes in the GP_CDS dataset.

### 2.4. Distribution Patterns of ENC Deviations

**Figure 1:**
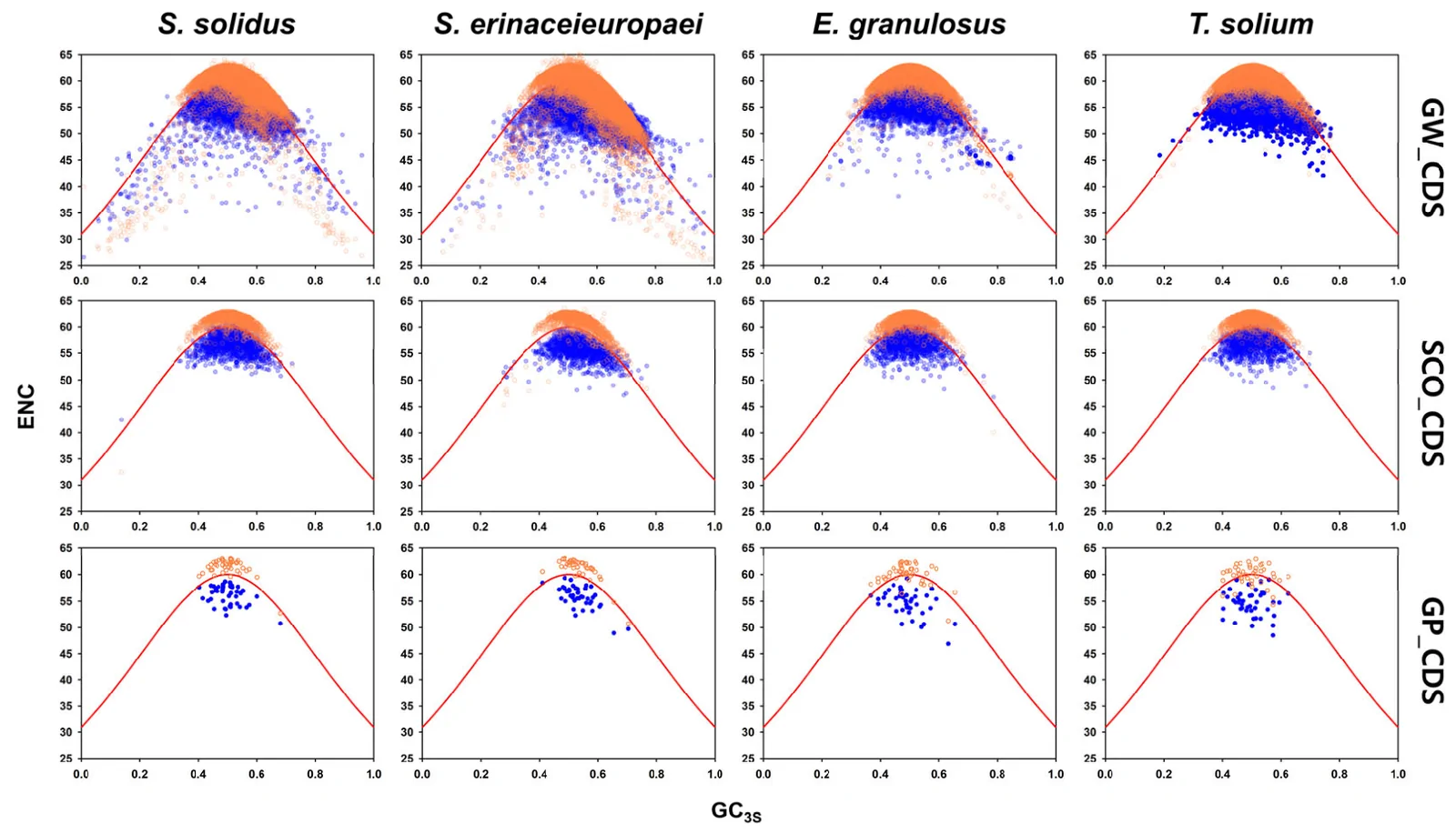
Relationships between GC_3S_ and the effective number of codons (ENC). Scatter plots show the relationships between GC_3S_ and observed ENC values in the GW_CDS, SCO_CDS, and GP_CDS datasets of the four cestode species. Solid blue and open orange circles indicate observed ENC values and DAMBE-derived expected ENC values, respectively. The red solid curve represents the theoretical ENC expected under Wright’s model when codon usage is determined solely by GC_3S_ composition.

While interspecific differences in mean ENC_obs_ were modest, ENC_exp_ values calculated by DAMBE differed from those expected from Wright’s theoretical relationship (Figure 1). Consequently, the residual ENC_obs_ deviations from Wright’s ENC_exp_ (ΔENC_W) or DAMBE’s ENC_exp_ (ΔENC_D) were analyzed to determine whether this variance reflects differences in codon usage patterns (Table 1 and Supplementary Figure S3). In the GW_CDS dataset, both ΔENC measures showed significant heterogeneity in dispersion among species (Fligner–Killeen tests, *p* < 0.001), although the magnitude of the test statistic was substantially greater for ΔENC_D (*χ*^2^ = 643.56) than for ΔENC_W (*χ*^2^ = 33.66). In the SCO_CDS dataset, significant heterogeneity was detected for ΔENC_D (*χ*^2^ = 16.69, *p* < 0.001) but not for ΔENC_W (*p* = 0.385). In contrast, the GP_CDS dataset showed significant heterogeneity for ΔENC_W (*χ*^2^ = 11.86, *p* = 0.007), whereas no significant difference was detected for ΔENC_D (*p* = 0.874). This suggests that the two models differ in their sensitivity to residual variation in the highly constrained GP_CDS dataset.

### 2.5. Relationships Between CDS Length and ENC Deviation

**Figure 2:**
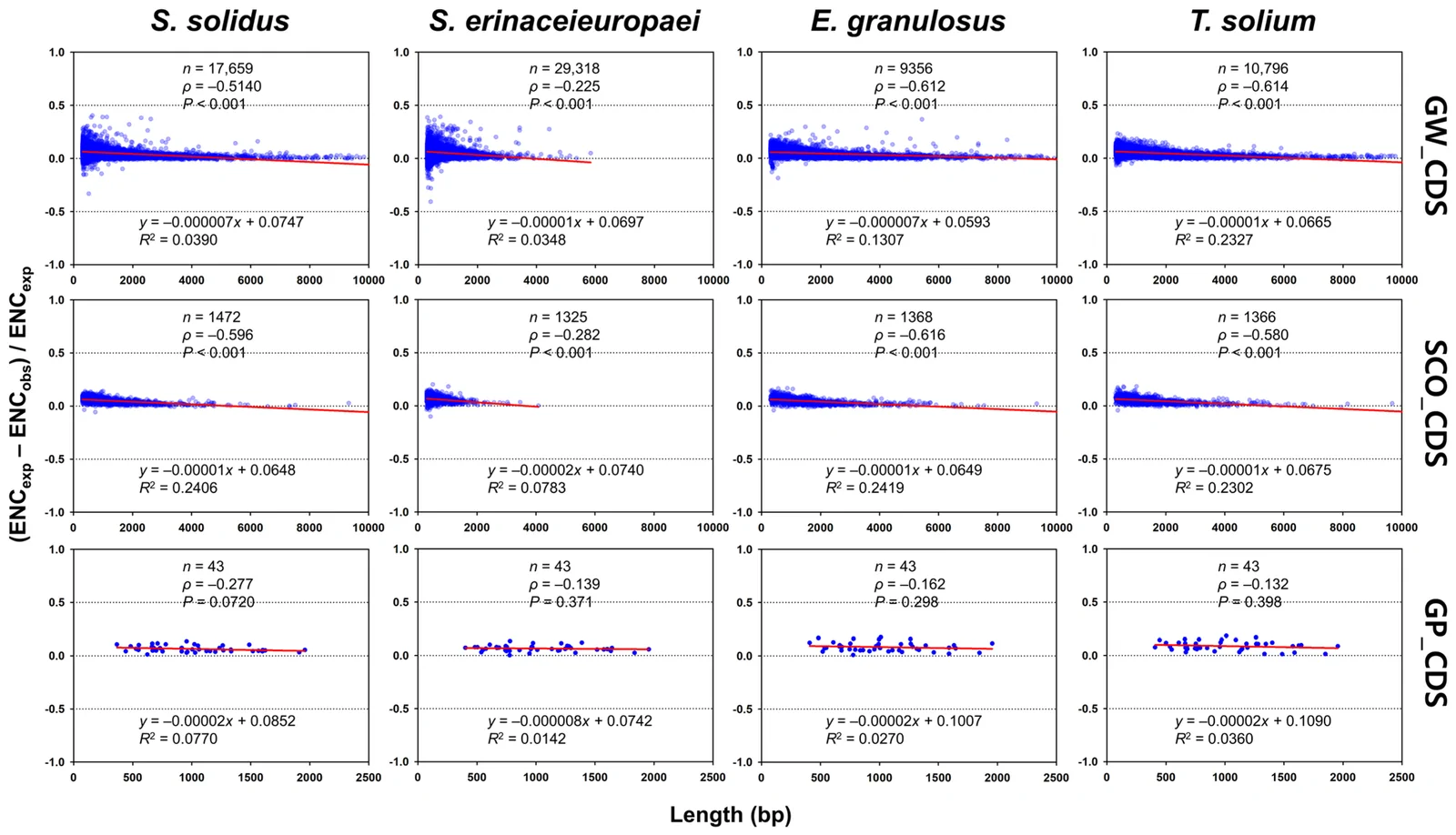
Relationships between CDS length and relative ENC deviation. Scatter plots show the relationships between CDS length and relative ENC deviation, calculated as (ENC_exp_ − ENC_obs_)/ENC_exp_, in the GW_CDS, SCO_CDS, and GP_CDS datasets, with ENC_exp_ calculated using Wright’s formula. Spearman’s correlation coefficients (ρ) and associated *p* values are shown together with sample sizes (*n*) in each panel. Red solid lines represent linear regression fits, with the corresponding regression equations and *R*^2^ values shown in each panel.

The ΔENC_W values exhibited moderate inverse associations with CDS length in the GW_CDS and SCO_CDS datasets of *S. solidus*, *E. granulosus*, and *T. solium* (Spearman rank-order correlation analysis, −0.614 ≤ ρ ≤ −0.514), whereas these relationships were substantially weaker in the corresponding GP_CDS datasets (−0.277 ≤ ρ ≤ −0.132). Linear regression analyses indicated that these associations were not adequately described by a linear model (*R*^2^ < 0.242; Figure 2). In contrast, non-parametric locally weighted scatterplot smoothing (LOESS) revealed high variability in relative ΔENC_W among shorter CDSs, which progressively decreased with increasing CDS length. The ΔENC_D values showed similar length-dependent patterns, although their absolute ρ values were generally somewhat lower (Supplementary Figure S4). Notably, the GW_CDS and SCO_CDS datasets of *S. erinaceieuropaei* showed only weak correlations (|ρ| < 0.3), despite their statistical significance (*p* < 0.001), suggesting that the statistical significance largely reflects the large sample sizes rather than strong biological associations.

Analysis of ΔENC dispersion further showed substantially greater variability among shorter CDSs, with the range of ENC deviation generally narrowing as CDS length increased. Heterogeneity in ΔENC dispersion across length classes was statistically confirmed by the Fligner-Killeen test (*p* < 0.001), indicating significant length-associated heterogeneity in ENC deviation. To account for this length dependence, CDSs were stratified into six length classes (300–599, 600–899, 900–1199, 1200–1499, 1500–1799, and ≥1800 bp), and the absolute magnitude of ΔENC_W (|ΔENC_W|) was compared among species within each class. Significant interspecific differences persisted across all six length classes in the GW_CDS dataset (Kruskal-Wallis tests, *p* < 3.0 × 10^−6^ in all classes), whereas significant differences in the SCO_CDS dataset were detected only in the shortest (300–599 bp, *p* = 0.004) and longest (≥1800 bp, *p* = 0.039) classes.

These results indicate that CDS length strongly influences the dispersion of ΔENC. However, the persistence of significant interspecific differences in |ΔENC_W| across all GW_CDS length classes indicates that differences in CDS length alone cannot account for the genome-wide interspecific variation. In the more conserved SCO_CDS dataset, these differences were largely attenuated and remained detectable only in the shortest and longest length classes, suggesting that increased gene comparability substantially reduces the heterogeneity in ΔENC observed in genome-wide datasets.

### 2.6. Base Composition and Codon Usage

**Figure 3:**
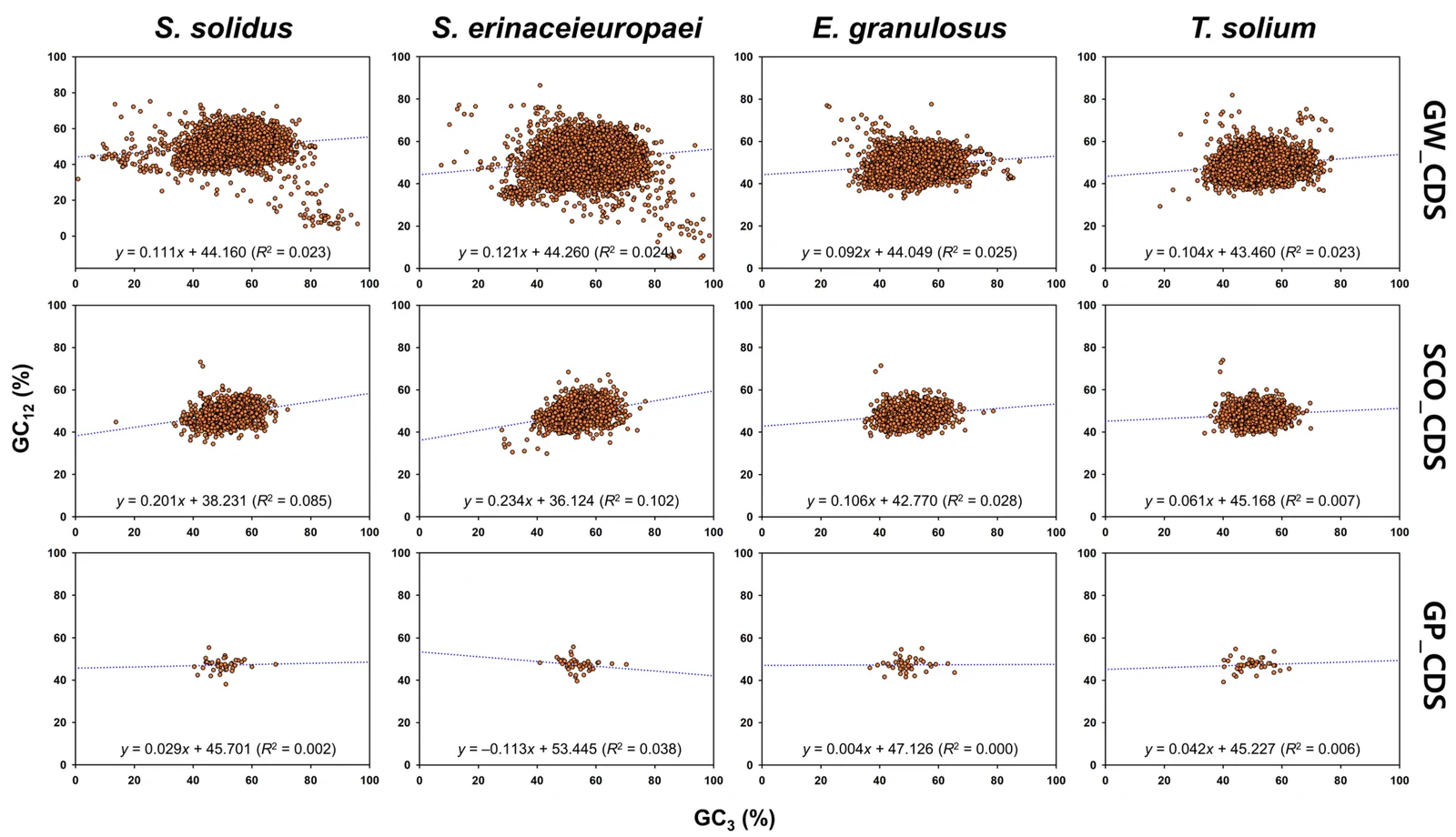
Neutrality plots of nucleotide composition across cestode CDS datasets. The relationships between GC_12_ and GC_3_ are shown for the GW_CDS, SCO_CDS, and GP_CDS datasets of the four cestode species. Dotted blue lines represent linear regression fits, with the corresponding regression equations and *R*^2^ values shown in each panel.

To identify the biological drivers underlying the residual variation in ΔENC, the influence of nucleotide composition was examined through neutrality, Parity Rule 2 (PR2) bias, and GC skew analyses. Neutrality plots of the GW_CDS dataset demonstrated low regression slopes (0.092–0.121) with limited explanatory power, as evidenced by low *R*^2^ values (0.023–0.025), indicating that variation in GC_3_ was only weakly reflected in GC_12_ (Figure 3). This pattern became more pronounced in the SCO_CDS and GP_CDS datasets, where slopes approached zero.

**Figure 4:**
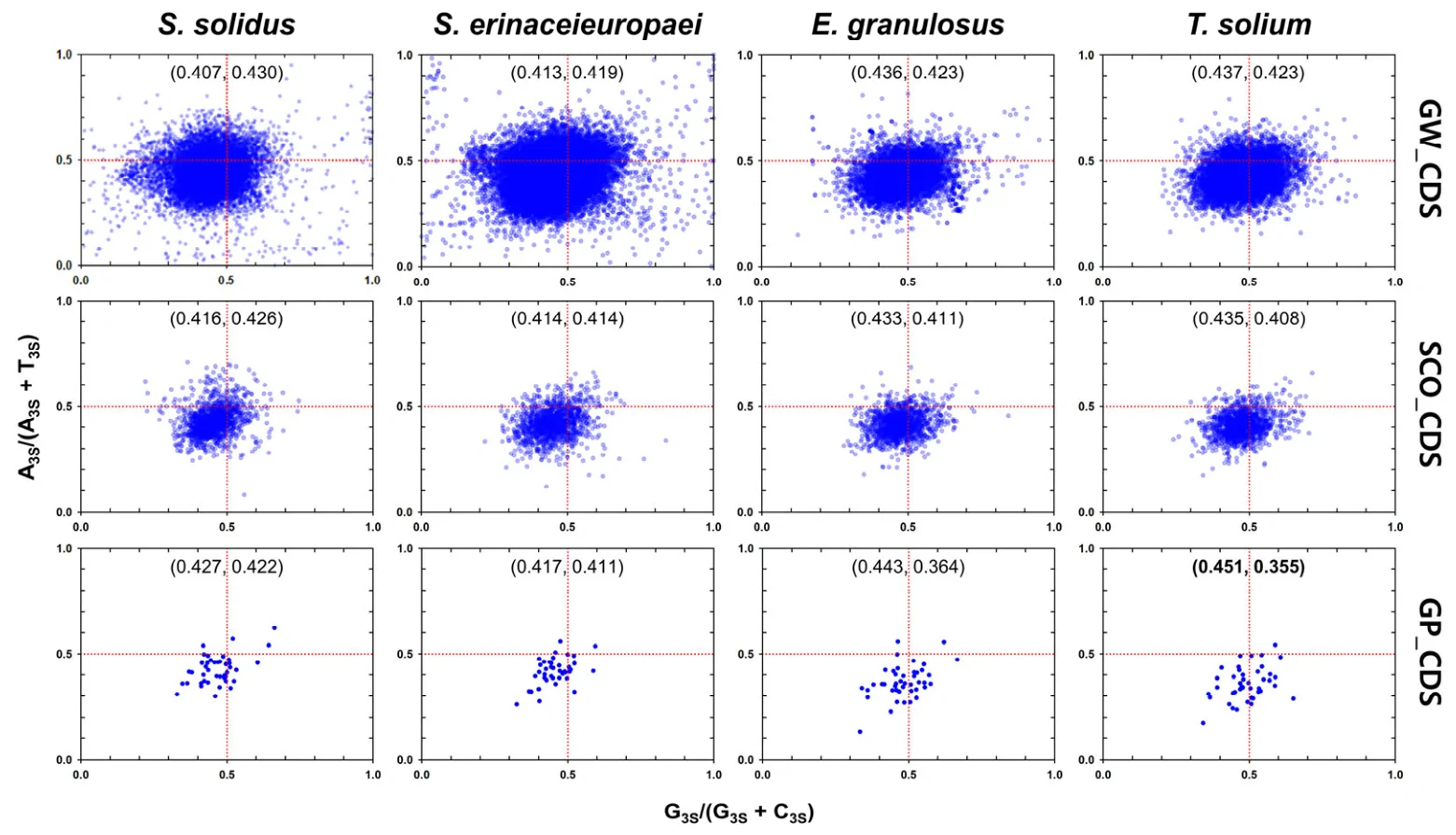
Parity Rule 2 (PR2) analysis of synonymous codon usage. A_3S_/(A_3S_ + T_3S_) values were plotted against G_3S_/(G_3S_ + C_3S_) values at fourfold degenerate codon positions in the GW_CDS, SCO_CDS, and GP_CDS datasets of the four cestode species. The red dotted lines intersecting at the center point (0.5, 0.5) indicate equal usage of complementary nucleotides. The coordinate values in parentheses represent the mean values for each dataset.

Although neutrality plots indicated weak correspondence between GC_3_ and GC_12_, PR2 bias analysis revealed a distinct genome-wide compositional asymmetry at the third codon position (Figure 4). The centroids for all species were located in the lower-left quadrant of the PR2 plot, indicating biases toward thymine and cytosine (A_3S_/[A_3S_ + T_3S_], 0.355–0.430; G_3S_/[G_3S_ + C_3S_], 0.407–0.451), where A_3S_, T_3S_, G_3S_, and C_3S_ represent the frequencies of adenine, thymine, guanine, and cytosine, respectively, at the third positions of synonymous codons. Thus, this compositional bias may represent a background influence on synonymous codon choice. Finally, relationships between strand-specific mutational bias (GC skew) and deviation from expected ENC (ΔENC ratio) revealed only a weak association between these variables (Supplementary Figures S5 and S6). Across species and datasets, Spearman rank-order correlation coefficients (ρ) were low in magnitude, although their direction varied among datasets and between methods used to estimate ENC_exp_. These weak associations were observed using both Wright- and DAMBE-derived ENC_exp_ values, suggesting that the magnitude of ENC deviation was only weakly associated with strand-specific mutational forces.

### 2.7. Comparison of Relative Synonymous Codon Usage Among Species

**Figure 5:**
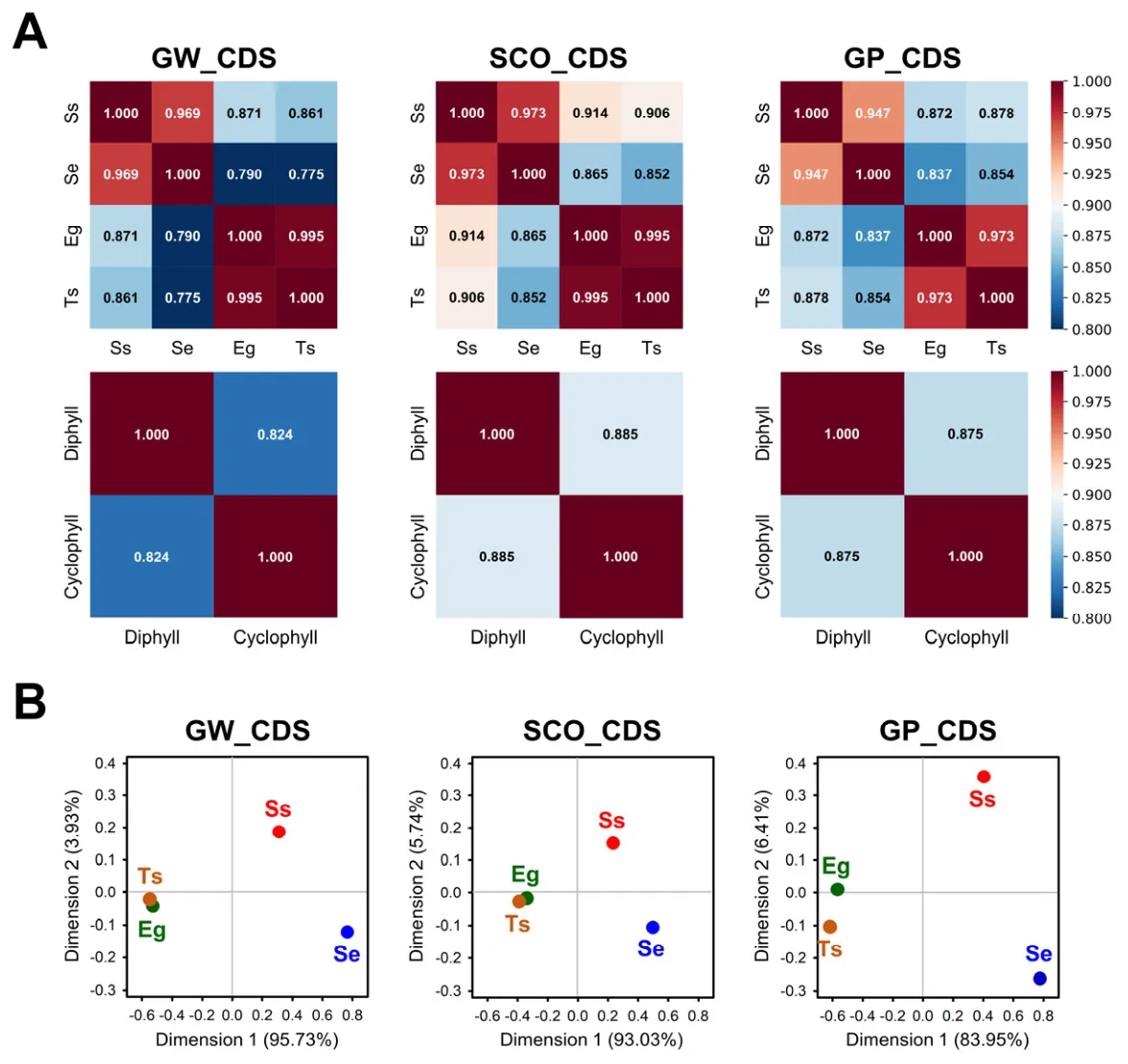
Correlation and correspondence analyses of relative synonymous codon usage (RSCU) across cestode CDS datasets. (**A**) Correlation matrices showing pairwise similarities in RSCU profiles among species (**upper** panels) and between lineages (**lower** panels) within the GW_CDS, SCO_CDS, and GP_CDS datasets. Values indicate Spearman rank-order correlation coefficients. Cell colors represent the values of the correlation coefficients according to the color scale shown on the right. (**B**) Correspondence analysis plots based on RSCU profiles of the four cestode species. Percentages in the axis titles indicate the proportions of total variation explained by Dimensions 1 and 2. Ss, *Schistocephalus solidus*; Se, *Spirometra erinaceieuropaei*; Eg, *Echinococcus granulosus*; Ts, *Taenia solium*.

Cestode species exhibited a consistent bias toward C- and T-ending codons, resulting in superficially similar preferred (relative synonymous codon usage [RSCU] ≥ 1.0) and strongly preferred (RSCU ≥ 1.2) codon profiles (Supplementary Table S2). However, statistical analysis revealed significant differences in the usage intensity of these C-ending (one-way ANOVA, *F* = 13.12, *p* < 0.001) and T-ending (*F* = 5.31, *p* < 0.01) subgroups across species. Variance partitioning analysis demonstrated that the majority of the observed variation (~76%) was attributable to the divergence between the Diphyllobothriidea and Cyclophyllidea, whereas only a minor fraction (~24%) was due to intra-lineage differences. Specifically, the diphyllobothriidean lineage showed a significantly stronger preference for C-ending codons (mean RSCU: 1.18 vs. 1.07; *p* < 0.00001), while the cyclophyllidean lineage favored T-ending variants (mean RSCU: 1.19 vs. 1.07; *p* < 0.001). These high levels of intra-lineage similarity were strongly supported by correlation and correspondence analyses (Figure 5), indicating that the two sampled lineage groups exhibit contrasting preferences for C-ending and T-ending synonymous codons, hereafter referred to descriptively as the “C-dialect” and “T-dialect,” respectively. These patterns remained robust across all three datasets, with the GP_CDS dataset exhibiting the most pronounced inter-lineage variation (Figure 5A).

Correspondence analysis further revealed that variation in codon usage was strongly associated with nucleotide identities at the third codon position, particularly C versus T (Dim1), which accounted for 95.73% of the total variance and clearly separated GC-biased diphyllobothriideans from AT-biased cyclophyllideans (Figure 5B and Supplementary Figure S7). These lineage-specific signatures are characterized by distinct preferences for specific synonymous codons, such as GTC (Val) and TCC (Ser) in diphyllobothriideans versus GCT (Ala) and GGT (Gly) in cyclophyllideans that remain consistent across codons decoding amino acids with diverse biochemical properties (Supplementary Figures S7 and S8; Supplementary Table S2). Notably, Dim2 also resolved intra-lineage divergence within the Diphyllobothriidea, driven by distinct preferences for codons such as GGC (Gly), GTC (Val), and CGC (Arg) in *S. erinaceieuropaei*, compared to GGT (Gly), GTA (Val), and TTA (Leu) in *S. solidus* (Figure 5B and Supplementary Table S2). The color-coded biochemical grouping of these codons further showed that these preferences extended across various amino acid classes, including nonpolar and polar/uncharged groups (Supplementary Figures S7 and S8). While Dim1, which primarily reflected differences in third-position nucleotide preferences, accounted for most of the variation in the GW_CDS and SCO_CDS datasets, its relative contribution decreased in the GP_CDS dataset, where Dim2 accounted for a greater proportion of variation (11.95%; Figure 5B). As illustrated by the codons mentioned above, differences in third-position nucleotide preferences may contribute to the additional variation represented by Dim2, particularly the separation between the two diphyllobothriidean species (Figure 5B). However, whether this pattern extends across the full set of synonymous codon families and what factors underlie this secondary variation remain unresolved.

### 2.8. Optimal Codons and Their Frequencies in Coding DNA Sequences

The strictly preferred optimal codon set defined for each CDS dataset revealed lineage-specific divergence in synonymous codon usage patterns (Supplementary Table S2). The distribution of optimal codons according to third-position nucleotide identity, particularly C- vs. T-ending codons, differed significantly between diphyllobothriideans and cyclophyllideans in the GW_CDS and SCO_CDS datasets under the single-optimal codon definition (Fisher’s exact tests, *p* = 0.0003 and 0.0033, respectively), whereas no significant difference was detected in the GP_CDS dataset (*p* = 0.1030; Supplementary Table S3). Binomial tests further supported lineage-specific directional biases, demonstrating significant enrichment of C-ending codons in the diphyllobothriidean GW_CDS and SCO_CDS datasets (two-sided tests, *p* = 2.16 × 10^−7^ and 0.0125, respectively) and T-ending codons in the cyclophyllidean GP_CDS dataset (*p* = 0.0052). The cyclophyllidean SCO_CDS dataset also showed a tendency toward T-ending codons, although this pattern was not statistically significant (*p* = 0.1360). In contrast, such directional preferences were not apparent under the multiple-optimal codon definition (RSCU ≥ 1), in which both C- and T-ending codons were frequently classified as preferred within the same synonymous codon families, thereby obscuring lineage-specific biases (Supplementary Tables S2 and S3).

**Figure 6:**
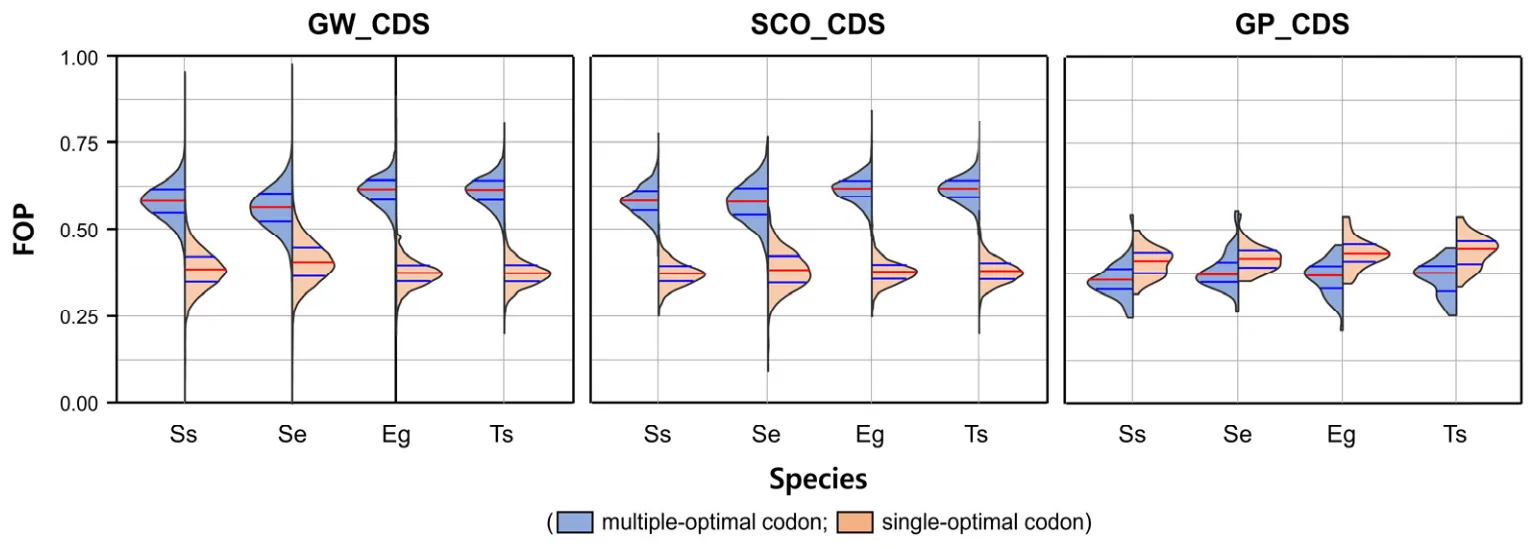
Distributions of the frequency of optimal codons (FOP) across cestode CDS datasets. Split violin plots show the distributions of FOP values calculated using multiple-optimal codon (blue) and single-optimal codon (orange) definitions across the GW_CDS, SCO_CDS, and GP_CDS datasets. Red and blue horizontal lines indicate the median and the first and third quartiles (Q1 and Q3), respectively. Species are grouped according to diphyllobothriidean (Ss and Se) and cyclophyllidean (Eg and Ts) lineages. Violin widths were normalized to facilitate comparison of distribution shapes independently of the number of CDSs. Ss, *Schistocephalus solidus*; Se, *Spirometra erinaceieuropaei*; Eg, *Echinococcus granulosus*; Ts, *Taenia solium*.

To further characterize the contrasting codon preferences between the two cestode lineages, the frequency of optimal codons (FOP) was calculated using both multiple- and single-optimal codon definitions across the three CDS datasets, and their distributions were compared among species (Figure 6 and Supplementary Table S4). Under the multiple-optimal codon definition, FOP values were significantly lower in the GP_CDS dataset than in the GW_CDS and/or SCO_CDS datasets across species (Wilcoxon rank-sum tests with Benjamini-Hochberg false discovery rate (FDR) correction, adjusted *p* < 0.001 for all GW vs. GP and SCO vs. GP comparisons; Supplementary Table S5). At the lineage level, these FOP values differed significantly between cyclophyllideans and diphyllobothriideans in the GW_CDS and SCO_CDS datasets (adjusted *p* < 0.001), but not in the GP_CDS dataset (adjusted *p* = 0.909; Supplementary Table S5).

Under the single-optimal codon definition, FOP values increased in the GP_CDS dataset relative to the GW_CDS and/or SCO_CDS datasets, with particularly strong and consistent increases in *E. granulosus* and *T. solium*. In these cyclophyllidean species, FOP values in the GP_CDS dataset were significantly higher than those in the GW_CDS and SCO_CDS datasets (adjusted *p* < 0.001 for all comparisons; Supplementary Tables S4 and S5). Trend analyses using the Jonckheere-Terpstra test further confirmed significant monotonic increases across datasets (GW_CDS → SCO_CDS → GP_CDS; adjusted *p* < 0.001 for both species; Supplementary Table S5). The FOP values in the GP_CDS dataset were also higher than those in the SCO_CDS dataset (adjusted *p* < 0.001) in diphyllobothriidean species. However, the increase relative to the GW_CDS dataset was weaker or not significant (*S. solidus*, adjusted *p* = 0.008; *S. erinaceieuropaei*, adjusted *p* = 0.320), and the overall trend remained non-monotonic. Pairwise interspecific comparisons revealed generally larger FOP effect sizes between species from different lineages than between those within the same lineage, although their magnitudes varied across CDS datasets and optimal-codon definitions (Supplementary Table S5). Importantly, single-optimal codon-based FOP values were significantly higher in cyclophyllideans than in diphyllobothriideans in the GP_CDS dataset (adjusted *p* < 0.001), whereas multiple-optimal codon-based FOP values showed no corresponding lineage-level difference. These results indicate that single-optimal codon-based FOP more effectively distinguishes optimal-codon usage patterns shared by each lineage group than multiple-optimal codon-based FOP, particularly the pronounced use of T-ending optimal codons in the highly curated cyclophyllidean genes.

### 2.9. Amino Acid and Codon Frequency Profiles

**Figure 7:**
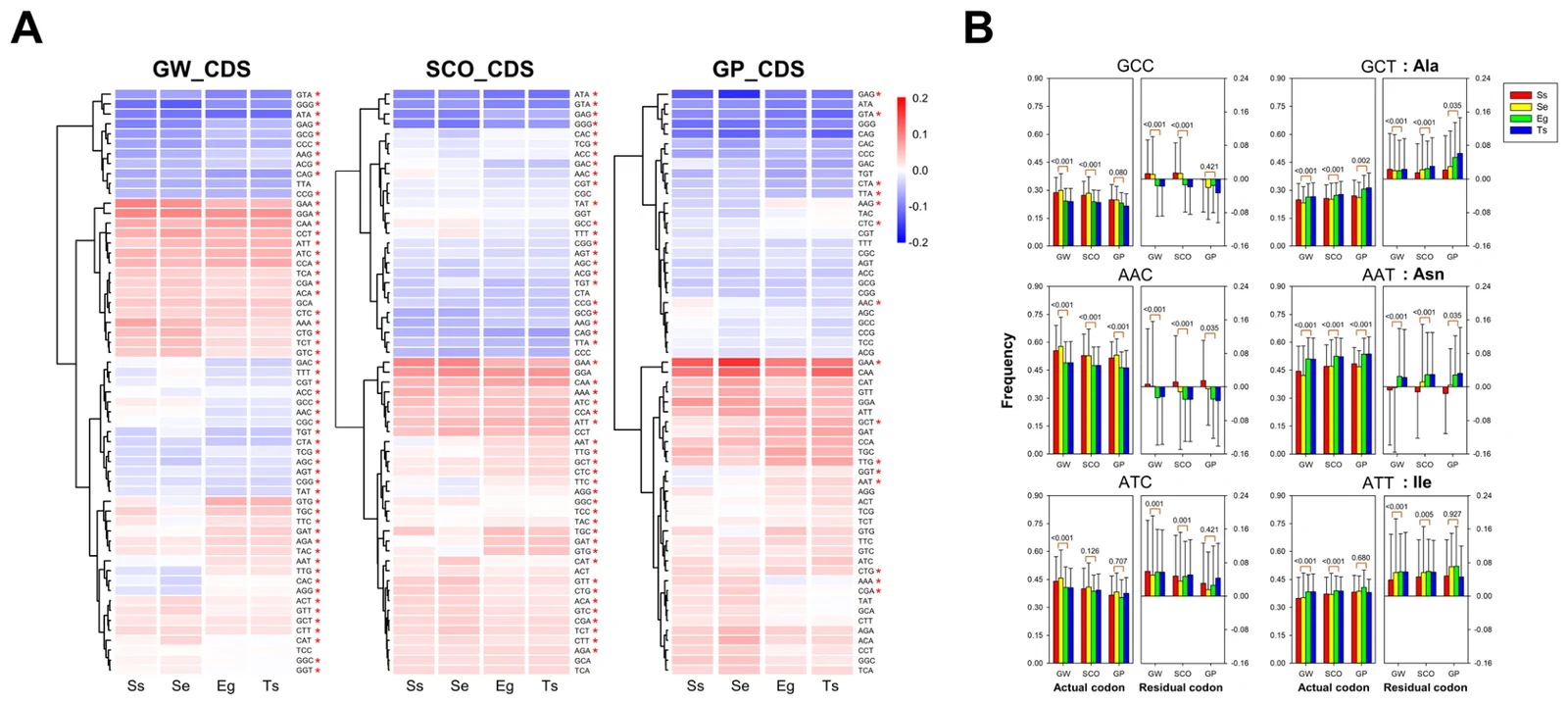
Lineage-associated restructuring of synonymous codon usage revealed by GC_3S_-corrected residual codon frequencies. (**A**) Heatmaps showing mean GC_3S_-corrected residual codon frequencies across the GW_CDS, SCO_CDS, and GP_CDS datasets. Cell colors represent residual codon frequency values according to the color scale shown on the right, with blue and red indicating negative and positive residuals, respectively. Asterisks indicate codons exhibiting significant differences between diphyllobothriidean and cyclophyllidean lineages based on Wilcoxon rank-sum tests with Benjamini-Hochberg false discovery rate (FDR) correction (FDR-adjusted *p* < 0.05). (**B**) Detailed comparisons of representative synonymous codon pairs, GCC/GCT (Ala), AAC/AAT (Asn), and ATC/ATT (Ile), across diphyllobothriidean (Ss and Se) and cyclophyllidean (Eg and Ts) species. Bars show mean actual codon frequencies (left subpanels) and mean GC_3S_-corrected residual codon frequencies (right subpanels), with error bars indicating standard deviations among genes. Values above brackets indicate FDR-adjusted *p* values from Kruskal–Wallis tests for interspecific differences. Ss, *Schistocephalus solidus*; Se, *Spirometra erinaceieuropaei*; Eg, *Echinococcus granulosus*; Ts, *Taenia solium*.

To further examine whether the lineage-associated optimal codon patterns described above were reflected in actual coding sequence composition, amino acid and codon frequency profiles were analyzed at the per-gene level in all three CDS datasets. Amino acid frequencies remained broadly conserved across lineages and exhibited only limited variation, whereas synonymous codon frequencies displayed substantially stronger and more heterogeneous lineage-associated shifts (Supplementary Figure S9). These lineage-associated differences were consistently observed in all three CDS datasets (Figure 7A), although their magnitude and distinctness varied considerably among synonymous codon families according to codon degeneracy structure (Figure 7B). The clearest reciprocal patterns were observed in strict two-fold degenerate codon families, particularly the AAC/AAT (Asn) pair, in which reciprocal enrichment of C- and T-ending codons produced highly distinct lineage-specific distributions with minimal overlap between diphyllobothriideans and cyclophyllideans. In contrast, synonymous codon pairs embedded within higher-degeneracy systems, such as the ATC/ATT (Ile) and GCC/GCT (Ala) pairs, exhibited comparatively weaker or more diffuse lineage separation patterns. Although the ATC/ATT pair retained clear lineage-associated directional trends, separation between codon distributions was less discrete than that observed for the AAC/AAT pair, potentially reflecting buffering effects associated with additional synonymous alternatives within multi-fold degenerate codon systems (Figure 7B).

Despite this increased dispersion, statistical analyses demonstrated that lineage-associated codon usage shifts remained highly significant across substantial fractions of synonymous codons (~100% in GW_CDS, ~83% in SCO_CDS, and ~22% in GP_CDS; Wilcoxon rank-sum tests with Benjamini-Hochberg FDR correction, adjusted *p* < 0.05; Supplementary Table S6). Notably, the C-ending codons GCC, AAC, GAC, ATC, and ACC consistently exhibited positive residual enrichment in diphyllobothriidean genomes, whereas their corresponding T-ending synonymous counterparts GCT, AAT, GAT, ATT, and ACT showed reciprocal enrichment in cyclophyllideans. These opposing residual patterns within synonymous codon families indicate that the observed restructuring of codon usage cannot be explained solely by compositional bias associated with GC_3S_. The persistence of these codon-specific residual shifts across both orthologous gene sets (SCO_CDS and GP_CDS datasets) and the whole coding-sequence dataset (GW_CDS dataset) further suggests that the observed codon-usage divergence extends beyond background compositional constraints.

### 2.10. Comparison of Codon Frequencies with tRNA Gene Repertoires

Prediction of genomic tRNA genes revealed substantial differences in repertoire size among the cestode species examined (164–9728 predictions), primarily due to wide variation in pseudogene proportions (8.632–89.280%; Supplementary Table S7). The number of functional tRNA genes also varied considerably in these species (135–1993 genes), with *T. solium* exhibiting an exceptionally reduced repertoire (135 genes). To facilitate interpretation of this reduced repertoire, the genomes of *Taenia saginata* and *Taenia asiatica* were additionally analyzed as comparative representatives of the genus *Taenia*. Both species possessed substantially smaller functional tRNA and pseudogene repertoires than *E. granulosus*, but larger repertoires than *T. solium*. These observations indicate that considerable diversification of genomic tRNA repertoires has occurred during species-level evolution within cestodes. Despite this variation in repertoire size, Spearman rank-order correlation analysis of 61 individual cognate tRNA profiles revealed a clear pattern of lineage-level conservation. Correlations between diphyllobothriidean and cyclophyllidean species were weak (ρ < 0.337; *p* > 0.009), whereas highly significant correlations were observed within both the diphyllobothriidean (ρ = 0.778; *p* = 2 × 10^−7^) and cyclophyllidean (ρ = 0.645; *p* = 5.85 × 10^−10^) lineages. Consistent with their taxonomic affiliation, *T. saginata* and *T. asiatica* exhibited much stronger correlations with *T. solium* (ρ = 0.822 and 0.732; *p* = 2 × 10^−7^) and *E. granulosus* (ρ = 0.549 and 0.433; *p* = 8.19 × 10^−6^ and 6.66 × 10^−4^, respectively) than with diphyllobothriidean species (ρ < 0.320; *p* > 0.078).

Potential associations between codon usage patterns and genomic tRNA gene repertoires were evaluated by comparing the functional tRNA gene counts predicted by tRNAscan-SE with corresponding codon frequencies (Supplementary Table S8). Overall relationships between these variables were generally weak, as indicated by low Spearman correlation coefficients (−0.143 ≤ ρ ≤ 0.168, 0.203 ≤ *p* ≤ 0.839; −0.164 ≤ ρ ≤ 0.201, 0.126 ≤ *p* ≤ 0.540; and −0.141 ≤ ρ ≤ 0.227, 0.084 ≤ *p* ≤ 0.814 for the GW_CDS, SCO_CDS, and GP_CDS datasets, respectively). These weak overall correlations suggested that different synonymous codon families might exhibit contrasting codon-tRNA relationships. To test this possibility, representative two-fold synonymous codon families were analyzed individually and classified according to the consistency of codon and tRNA preference patterns across the CDS datasets (Table 2). This analysis revealed substantial heterogeneity among codon families. The Cys and Glu families exhibited strong concordance, with lineage-associated codon preferences closely matching corresponding tRNA gene repertoires. In contrast, the Asn and Gln families displayed persistent codon-tRNA opposition across all datasets and were assigned to the strong-discordance category. Several additional codon families, including Asp, His, Phe, Tyr, and Lys, exhibited intermediate patterns characterized by moderate concordance or discordance. Among these families, Asn showed the strongest overall discordance between codon and tRNA preferences, whereas Cys exhibited the most pronounced concordance. The Gln family similarly showed persistent codon-tRNA opposition despite substantial lineage-associated divergence in tRNA gene abundance, while the Glu family demonstrated strong concordance accompanied by large differences in cognate tRNA gene counts.

Collectively, these findings reveal lineage-associated differences in genomic tRNA repertoires alongside the contrasting codon usage patterns observed in the examined cestodes. However, the relationships between codon usage and corresponding tRNA gene repertoires are highly family-specific, with both concordant and discordant evolutionary patterns observed among different synonymous codon families. The coexistence of these contrasting patterns likely underlies the weak overall correlations between codon frequencies and cognate tRNA gene abundances, indicating that codon usage and tRNA repertoire evolution are not universally coupled across the genome.

## 3. Discussion

In the present study, synonymous codon usage patterns were systematically investigated in representative diphyllobothriidean and cyclophyllidean cestodes using the GW_CDS and SCO_CDS datasets at the genome-wide and SCO levels, respectively. The GP_CDS dataset, constructed based on orthologous relationships among proteins retrieved from GenBank, exhibited relatively homogeneous CDS lengths and was further analyzed to minimize potential biases arising from CDS truncation and annotation errors in the GW_CDS and SCO_CDS datasets, which exhibited substantial interspecific variation in CDS length. Although overall codon usage bias was relatively weak and broadly similar among species, as indicated by ENC analyses, multiple complementary approaches, including RSCU and correspondence analyses, consistently revealed pronounced lineage-associated divergence in synonymous codon preferences. Diphyllobothriidean genomes preferentially utilized C-ending synonymous codons, whereas cyclophyllidean genomes exhibited corresponding enrichment of T-ending alternatives, forming distinct lineage-specific codon dialects. These patterns persisted across all CDS datasets and remained detectable after correction for GC_3S_-associated compositional effects, suggesting that factors beyond mutational bias may contribute to the observed divergence, despite interspecific differences in genomic and CDS GC contents and the strong association between GC_3S_ and the principal dimension of correspondence analysis. Neutrality plot, PR2, and GC skew–ΔENC analyses further indicated the involvement of non-compositional factors in shaping the observed codon usage patterns. Notably, lineage-associated divergence was substantially more pronounced at the synonymous codon level than at the amino-acid level, suggesting that the observed evolutionary divergence was primarily reflected in synonymous codon usage rather than amino-acid composition. Comparative analyses of genomic tRNA repertoires further revealed clear lineage-associated differences in tRNA gene composition. However, the relationships between codon usage and corresponding tRNA gene abundances varied substantially among synonymous codon families, with both concordant and discordant patterns observed. Collectively, these findings suggest that lineage-associated divergence in synonymous codon usage may reflect broader differences in translational architectures among cestodes, although the relationships between codon usage and genomic tRNA repertoires remain heterogeneous across individual codon families.

The GW_CDS and SCO_CDS datasets used in this study were prepared using CDSs predicted from whole-genome assemblies that exhibited markedly different levels of contiguity (approximate scaffold N50 values of 31.5 kb, 4.6 kb, 18.7 Mb, and 67.8 kb in the *S. solidus*, *S. erinaceieuropaei*, *E. granulosus*, and *T. solium* genomes, respectively). Identification of protein-coding genes from low-N50 draft assemblies frequently results in inaccurate gene architectures, generating truncated or split coding sequences and consequently altering apparent gene numbers and CDS lengths [24,25,26]. The unusually large number and short median length of *S. erinaceieuropaei* CDSs are therefore likely attributable to the highly fragmented nature of the available genome assembly (Table 1). Similar, although less pronounced, effects may also be present in the *S. solidus datasets*. This interpretation is further supported by the exceptionally high frequencies of non-ATG-initiating CDSs in the *S. erinaceieuropaei* (28.29%) and *S. solidus* (18.67%) GW_CDS datasets, compared with *E. granulosus* (0.00%) and *T. solium* (3.16%), as well as by the elevated proportions of CDSs lacking canonical initiation and/or termination signals (Supplementary Table S9). Given that codon usage patterns may differ among 5′-terminal, internal, and 3′-terminal coding regions [27,28], truncation and fragmentation of CDSs could potentially distort codon usage estimates by disproportionately sampling localized sequence segments with distinct compositional properties. This effect is likely compounded by the greater statistical variance and reduced precision of codon-bias indices calculated from short coding sequences [29,30]. Consistent with this interpretation, ΔENC values exhibited substantially greater dispersion among short CDSs and progressively converged toward zero as CDS length increased, whereas these relationships were markedly attenuated in the GP_CDS dataset (Figure 2 and Supplementary Figure S4). Nevertheless, length-stratified analyses indicated that factors other than CDS length also contribute to the observed interspecific variation in |ΔENC_W|. Collectively, these observations suggest that assembly fragmentation and the resulting abundance of short CDSs, especially in *S. erinaceieuropaei*, contribute to the dispersion and reduced precision of codon-bias estimates, but do not fully account for the observed interspecific variation in ΔENC.

In addition to the findings from the length-stratified ΔENC analyses, other evidence further indicates that CDS fragmentation alone cannot account for the principal codon-usage patterns identified in this study. The major codon-usage signatures remained detectable in the ortholog-restricted SCO_CDS and manually curated GP_CDS datasets despite progressive reduction of CDS length heterogeneity and likely truncation effects (Figure 2 and Figure 5, Figure 6 and Figure 7; Supplementary Figure S9). Moreover, *S. solidus* exhibited codon-usage characteristics highly similar to those of *S. erinaceieuropaei*, including the lineage-associated enrichment of C-ending synonymous codons, despite possessing a substantially more contiguous genome assembly and displaying ΔENC-length relationships comparable to those observed in *E. granulosus* and *T. solium*. If CDS fragmentation were the principal determinant of the observed codon-usage profiles, substantially greater divergence would be expected between the two diphyllobothriidean species. Instead, the conservation of codon-usage patterns across species and across progressively filtered CDS datasets suggests that the major codon-usage signatures identified here represent genuine characteristics of the examined species rather than assembly-derived artifacts. Although the principal lineage-associated patterns appear robust, future analyses based on high-contiguity *S. erinaceieuropaei* genome assemblies and improved gene annotations, such as those reported by Kikuchi et al. [14], will help further refine the quantitative estimates of codon usage bias in this species.

Genomic nucleotide composition appears to provide an important framework for codon-usage patterns [31]. The close relationships between genomic GC content and synonymous codon usage reported in a wide range of organisms support the contribution of mutational biases to codon-usage evolution [3,8,32,33]. In the cestodes examined here, the enrichment of GC content at the first and third codon positions relative to the genomic background, together with the weak correspondence between GC_12_ and GC_3_, suggests that compositional and position-specific constraints jointly influence coding-sequence organization, consistent with the differing functional constraints acting on individual codon positions [31,34,35] (Table 1; Figure 3). PR2 analysis [36] further revealed a common pyrimidine-biased compositional background, within which diphyllobothriideans exhibited relatively greater cytosine enrichment and cyclophyllideans greater thymine enrichment (Figure 4). These contrasting preferences were also reflected in correspondence analysis, where third-position nucleotide identity, particularly C versus T, largely distinguished the two cestode groups (Figure 5). Nevertheless, lineage-associated codon-frequency differences persisted after correction for GC_3S_, particularly for representative synonymous codon pairs such as AAC/AAT, GCC/GCT, and ATC/ATT (Figure 7B and Supplementary Table S6). Moreover, *S. solidus* exhibited codon-usage characteristics broadly similar to those of *S. erinaceieuropaei* despite possessing a genomic GC content closer to that of the cyclophyllidean species (Table 1). Collectively, these observations suggest that nucleotide composition provides an important background for codon-usage evolution, but does not fully explain the contrasting C- and T-ending codon preferences. Additional evolutionary constraints, potentially including selective factors, may therefore contribute to their emergence and maintenance.

Optimal codon profiles provided additional insights into the evolutionary significance of the lineage-specific codon dialects identified in this study (Figure 6; Supplementary Tables S2–S4). The more clearly structured optimal-codon patterns observed in the highly curated GP_CDS dataset may partly reflect the progressive reduction of technical and gene-associated heterogeneity across the GW_CDS, SCO_CDS, and GP_CDS datasets, allowing lineage-associated synonymous codon preferences to be more readily distinguished from background variation. Importantly, however, this effect was asymmetric between the two cestode lineages. Cyclophyllidean GP_CDS genes exhibited pronounced enrichment of specific T-ending optimal codons, accompanied by a significant increase in single-optimal codon-based FOP, whereas diphyllobothriideans showed comparatively modest and non-monotonic changes despite maintaining their baseline preference for C-ending synonymous codons (Supplementary Tables S2–S5). This asymmetry closely parallels the PR2 bias patterns, in which thymine enrichment remained prominent across all datasets in cyclophyllideans, whereas the relative cytosine enrichment observed in diphyllobothriideans became less pronounced in the SCO_CDS and GP_CDS datasets (Figure 4). This asymmetry may also be related to the intra-lineage divergence observed along Dim2 in correspondence analysis, where *S. erinaceieuropaei* and *S. solidus* exhibited contrasting preferences for representative C-ending and T/A-ending codons (Figure 5B). Thus, the contrasting C- and T-ending codon dialects do not appear to represent simple reciprocal consequences of background nucleotide composition. Rather, their differential behavior across progressively restricted CDS datasets suggests that the evolutionary constraints underlying these codon preferences may differ between the two lineages, with particularly pronounced enrichment of specific T-ending optimal codons in cyclophyllideans [37,38].

Given the broadly conserved amino-acid frequency profiles among cestode species (Supplementary Figure S9), the major evolutionary differences underlying codon-usage divergence appear to reside primarily within the synonymous-coding rather than protein-compositional layer of genome organization [3,8]. Nevertheless, several amino acids exhibited consistent directional differences between the two cestode lineages across the different CDS datasets (Supplementary Figure S9). These relatively subtle shifts showed no obvious correspondence with simple physicochemical or broad metabolic groupings, and their biological significance therefore remains unclear. Because synonymous codons encoding the same amino acid can differ in their interactions with the translational machinery, particularly through the availability and decoding properties of corresponding tRNAs, lineage-specific restructuring of the tRNA repertoire represents a plausible translational correlate of the contrasting codon dialects [4,6]. Comparative analyses of genomic tRNA genes indeed revealed substantial inter-lineage differences in both total functional tRNA gene numbers and anticodon compositions (Supplementary Tables S7 and S8). A marked reduction and restructuring of functional tRNA gene complements were observed in cyclophyllideans, particularly within taeniid species, together with the strong conservation of tRNA repertoire profiles within each cestode lineage, and considerable divergence in the genomic organization of tRNA repertoires between diphyllobothriideans and cyclophyllideans. These lineage-associated changes in genomic tRNA repertoires therefore provide a translational context for the evolution of contrasting synonymous codon preferences. However, whether codon usage and tRNA repertoires have evolved through direct and coordinated adaptation cannot be inferred from their parallel lineage-associated divergence alone. Indeed, analysis of individual synonymous codon families revealed substantially heterogeneous relationships between lineage-associated codon preferences and corresponding tRNA gene abundances (Table 2; Supplementary Table S8). Several codon families exhibited concordant changes in codon preference and cognate tRNA gene abundance, consistent with potential coordination between synonymous codon usage and the decoding machinery, whereas others showed weak or opposing relationships despite pronounced codon-usage divergence. The coexistence of these concordant and discordant patterns indicates that coupling between synonymous codon usage and genomic tRNA repertoires is codon-family-specific rather than globally uniform. Such heterogeneity may reflect differences in codon degeneracy and wobble-decoding properties, as well as tRNA expression, charging, and post-transcriptional modification, emphasizing that genomic tRNA gene copy numbers provide useful information about the potential genomic tRNA repertoire but do not directly measure functional translational capacity [39].

Taken together, the present results suggest that the contrasting C-ending and T-ending codon preferences may have emerged during the evolutionary divergence of diphyllobothriidean and cyclophyllidean cestodes, with the T-ending preference potentially becoming more pronounced during cyclophyllidean evolution, alongside genome condensation and changes in genomic tRNA repertoires. Although the generally lower genomic GC contents of cyclophyllideans provide a compositional background favoring T-ending codons, the maintenance of a pronounced C-ending codon preference in *S. solidus*, despite its relatively low genomic GC content, indicates that AT-biased nucleotide composition alone is insufficient to explain the emergence of the cyclophyllidean T-dialect. The marked reduction and restructuring of genomic tRNA repertoires observed particularly in taeniid species further raise the possibility that changes in synonymous codon usage accompanied broader remodeling of the translational machinery during cyclophyllidean evolution. Such an association is biologically plausible, since the relative availability of cognate tRNAs can influence codon-decoding rates and translational efficiency [5,6]. Beyond genome architecture, these lineage-specific codon dialects may also have broader biological significance, as synonymous codon usage and cellular tRNA pools can be differentially organized in relation to cellular proliferation and differentiation [7]. Whether these lineage-associated differences in codon usage and tRNA repertoires are related to the contrasting developmental and life-history strategies of cestodes remains an interesting question for future investigation, although the exceptional proliferative capacity of *S. proliferum* indicates that such relationships cannot be attributed simply to phylogenetic affiliation [14]. Importantly, the coexistence of concordant and discordant codon-tRNA relationships in the present study further indicates that any evolutionary coupling between codon usage and tRNA repertoires has operated in a codon-family-specific rather than globally uniform way. However, only two species from each lineage were examined in this study, and thus further comparative studies with broader taxonomic sampling, accompanied by functional characterization of their translational systems, are necessary to determine whether the contrasting CUB patterns are closely associated with the evolutionary divergence of these cestode lineages. The possible contributions of genome architecture, tRNA repertoire composition, wobble-decoding capacity, and life-history strategies to the observed codon-usage divergence also remain to be investigated.

## 4. Materials and Methods

### 4.1. Sequence Data

Genome sequence information for *E. granulosus* (BioProject PRJNA754835; assembly ASM2155672v1), *S. solidus* (PRJEB527; assembly S_solidus_NST_G2_0011_upd), *S. erinaceieuropaei* (PRJEB1202; assembly S_erinaceieuropaei), and *T. solium* (PRJNA170813; assembly Tsolium_Mexico_v1) was obtained from WormBase ParaSite (https://parasite.wormbase.org/species.html#Platyhelminthes, accessed on 20 November 2025). For each species, whole-genome sequences, CDSs, predicted protein sequences, and corresponding GFF3 gene annotation files were downloaded and used as inputs for orthology inference, codon usage analyses, and tRNA mapping.

A total of 1785 *S. erinaceieuropaei* protein sequences were retrieved from GenBank on 29 November 2025. This dataset comprised sequences identified either through experimental characterization of individual proteins or via BLAST-based annotation of expressed sequence tags. After removal of redundant entries and allelic variants, 129 non-redundant proteins were retained and used for subsequent ortholog screening in the other cestode species.

### 4.2. Coding DNA Sequence Datasets for CUB

To obtain high-confidence CDSs for codon-usage analyses, sequences shorter than 300 bp (100 codons) were filtered out from the raw CDS files downloaded from WormBase ParaSite. The remaining CDSs were designated as the GW_CDS set and used in the subsequent base composition and codon usage analyses. Since a substantial fraction of CDSs, particularly in *S. solidus* and *S. erinaceieuropaei*, initiated with alternative start codons rather than the canonical ATG, the presence of ATG at their 5′ ends was not imposed as a filtering criterion. Orthology relationships among the four GW_CDS sets were inferred using OrthoFinder ver. 3.0.1b1 [40]. The program was executed under a Conda-controlled environment (Conda ver. 25.9.1) using default similarity-search parameters. From the resulting orthogroups, those containing a single-copy ortholog from each of the four species were extracted to ensure one-to-one gene correspondence (SCO_CDS set).

Separately, amino acid sequences of the 129 GenBank *S. erinaceieuropaei* proteins were used as queries in BLAST searches against the GenBank proteomes of *E. granulosus*, *S. solidus*, and *T. solium* to retrieve their respective orthologs. Candidate orthologs were initially defined based on reciprocal best-hit relationships, considering BLAST score, query coverage, and sequence length, with the requirement that a single representative sequence be identified per species. In cases where multiple high-confidence matches were obtained for a single *S. erinaceieuropaei* protein, clustering patterns observed in BLAST-generated distance trees were additionally examined to infer the closest orthologous relationships. The evolutionary coherence of these BLAST-based orthogroups was further verified using OrthoFinder with candidate-supplemented complete proteomes of the four cestode species as input. Finally, CDSs corresponding to 43 protein tetrads showing concordant assignment of all member proteins to a single OrthoFinder orthogroup were retained for downstream analyses and defined as the GP_CDS set.

### 4.3. Calculation of Codon Usage Indices

For each CDS set, codons were analyzed according to the standard nuclear genetic code, excluding start and stop codons. The proportions of guanine (G) and cytosine (C) at the first (GC_1_), second (GC_2_), and third (GC_3_) codon positions were calculated for each CDS using the SeqinR package (ver. 4.2-36) [41] in R. To accurately assess CUB, the monocodonic Met (ATG) and Trp (TGG) codons were excluded from the counts. Values from synonymous positions were distinguished by the suffix ‘S’, resulting in metrics such as G_3S_ and GC_3S_. The ENC values were calculated for individual CDSs using DAMBE ver. 7.3.32 [42] with default settings. Observed ENC values (ENC_obs_) were obtained for each CDS to assess gene-level variation in codon usage. Expected ENC values (ENC_exp_) were obtained using two approaches: DAMBE-derived ENC_exp_ values corresponding to the GC_3S_ value of each CDS and ENC_exp_ values calculated from Wright’s theoretical relationship [43] across the full GC_3S_ range. Because the ENC_exp_ values obtained by the two approaches were not identical, both were retained for subsequent analyses. Deviations from expectation were calculated separately as ΔENC_D = DAMBE ENC_exp_ − ENC_obs_ and ΔENC_W = Wright ENC_exp_ − ENC_obs_. Synonymous codon-usage preferences were quantified using RSCU values, calculated via the *uco* function in the SeqinR package. For each species, in-frame CDSs were concatenated to generate representative coding sequences for the calculation of RSCU values across the 61 sense codons. To enhance the resolution for identifying species-specific codon preferences, the primary ‘optimal codon’ for each amino acid was strictly defined as the single codon exhibiting the highest RSCU value (‘single-optimal codon’ set) within each respective dataset. Additionally, for comparative analysis, a secondary, broader definition was applied, wherein multiple codons with an RSCU ≥ 1 were designated as optimal (‘multiple-optimal codon’ set). Both species-specific optimal codon sets were subsequently utilized to calculate FOP for individual genes. The resulting gene-level FOP values were compared among CDS datasets, species, and the two pooled lineage groups, as described in Section 4.4. This dual approach not only quantified the extent to which each sequence conforms to the predominant codon usage patterns of the corresponding species but also served to evaluate the impact of strict versus broad optimal codon criteria on the characterization of lineage-associated optimal codon usage patterns.

In order to evaluate the consistency of codon usage patterns between species and lineages, Spearman rank-order correlation coefficients were calculated based on the RSCU profiles. Furthermore, correspondence analyses were performed on the RSCU indices for the identification of the primary factors driving codon usage variation, after excluding the invariant codons for Met and Trp. This multivariate analysis was used to project species (observations) and codons (variables) onto a multi-dimensional space, where the major axes (Dimensions 1 and 2) represent the most significant sources of variation in the dataset. All statistical analyses and visualizations were conducted in the R environment.

### 4.4. Statistical Analysis of Codon Usage Drivers

Observed ENC values were plotted against GC_3S_ to examine the major determinants of CUB. Differences in the ENC_obs_-GC_3S_ relationship among species and between major cestode lineages were assessed using analysis of covariance (ANCOVA). A second-order polynomial regression (*y = ax^2^ + bx + c*) was applied to characterize the ENC_obs_-GC_3S_ relationship, and the interactions between polynomial GC_3S_ terms and lineage or species were used to assess divergence in these relationships. Deviations from expected ENC were quantified using the two measures defined above, ΔENC_W and ΔENC_D. Heterogeneity in their dispersion among species was evaluated using the Fligner-Killeen test, a non-parametric method robust to non-normal distributions and outliers common in large genomic datasets.

The relationships of ENC deviation with CDS length and GC skew ([G_3S_ – C_3S_]/[G_3S_ + C_3S_]) were examined using Spearman rank-order correlation and linear regression analyses. For analyses of CDS length, relative ENC deviations were calculated as (ENC_exp_ − ENC_obs_)/ENC_exp_ using Wright- and DAMBE-derived ENC_exp_ values, respectively. Non-linear relationships with CDS length were further characterized using LOESS. Heterogeneity in ΔENC dispersion across CDS length classes was evaluated using the Fligner-Killeen test. In addition, CDSs were stratified into predefined length intervals (300–599, 600–899, 900–1199, 1200–1499, 1500–1799, and ≥1800 bp), and interspecific differences in the absolute magnitude of ΔENC_W (|ΔENC_W|) within each class were evaluated using the Kruskal-Wallis test. Statistical significance was defined as *p* < 0.05. All analyses, including effect size estimation (partial eta squared, $\eta_{p}^{2}$, for ANCOVA and rank-biserial correlation for pairwise comparisons), were performed in R (ver. 4.5.0) using the car (ver. 3.1.5) and effectsize (ver. 1.0.2) packages. For the FOP comparisons, rank-biserial correlation coefficients were calculated to quantify the magnitude of differences between CDS distributions [44], and their absolute values (|*r*|) were presented alongside Benjamini–Hochberg-adjusted *p*-values.

Neutrality tests were performed by plotting GC_12_ against GC_3_ for individual CDSs to assess the relative contributions of mutation pressure and selective constraints to codon usage. Linear regression analysis was performed, and the slope of the regression line was interpreted as an estimate of the extent to which variation in GC_3_ is reflected in GC_12_. A slope approaching 1 indicates a predominant influence of mutation pressure, whereas a slope approaching 0 suggests stronger selective constraints. PR2 bias was evaluated at four-fold degenerate sites by calculating the nucleotide proportions A_3S_/(A_3S_ + T_3S_) and G_3S_/(G_3S_ + C_3S_). Under conditions of no bias, both ratios are expected to be 0.5. Deviations from the center point (0.5, 0.5) indicate unequal usage of complementary nucleotides, reflecting the influence of mutation bias and/or selective forces.

### 4.5. Analyses of Amino Acid and Codon Frequencies

Codon usage and amino acid frequency profiles were analyzed across the GW_CDS, SCO_CDS, and GP_CDS datasets using the standard genetic code with the SeqinR package in R. For each CDS, synonymous codon frequencies were calculated as the proportion of each codon relative to the total count of synonymous codons within its corresponding amino acid family, whereas amino acid frequencies were calculated relative to total amino acid counts within each CDS. To evaluate synonymous codon usage independent of background nucleotide composition, GC_3S_-corrected residual codon frequencies were additionally calculated, using GC_3S_ values estimated from synonymous third codon positions excluding monocodonic (Met and Trp) and stop-codon families. The six-fold degenerate codon families for Leu, Arg, and Ser were partitioned into independent synonymous subfamilies prior to analysis. Expected synonymous codon frequencies were estimated from per-gene GC_3S_ values under the neutral mutational model [43], and residual frequencies were calculated as the difference between observed and expected synonymous frequencies.

Per-gene amino acid, codon, and residual frequency distributions were summarized at species and lineage levels for statistical comparison and visualization. Frequency distributions between diphyllobothriidean and cyclophyllidean species were compared using Wilcoxon rank-sum tests, whereas interspecific comparisons were performed using Kruskal-Wallis tests. *p* values were adjusted across 59 synonymous codons using the Benjamini-Hochberg FDR correction.

### 4.6. Prediction and Comparative Analysis of tRNA Gene Repertoires

Transfer RNA genes were predicted from the genome assemblies of *S. solidus*, *S. erinaceieuropaei*, *E. granulosus*, and *T. solium* using tRNAscan-SE (ver. 2.0.12) with default eukaryotic parameters. Predicted loci were classified according to the annotation categories provided by the program, including functional tRNA genes, pseudogenes, suppressor tRNAs, and undetermined predictions. The numbers of functional tRNA genes, anticodon types, and pseudogenes were summarized for each species to characterize their genome-wide tRNA gene repertoires. To provide comparative data for the taeniid lineage, the genomes of *T. saginata* and *T. asiatica* available in GenBank (GCA_001693075.2 from PRJNA71493 and GCA_001693035.2 from PRJNA299871, respectively; [22]; accessed on 27 May 2026) were additionally analyzed using the same procedure. Functional tRNA gene counts corresponding to individual anticodons were extracted from tRNAscan-SE outputs and mapped to their decoded synonymous codons according to standard wobble-pairing rules.

Codon-specific tRNA gene counts were compared with synonymous codon frequencies for representative two-fold degenerate codon families. For each codon family, lineage-associated codon preferences were determined from mean codon frequencies calculated separately for diphyllobothriidean and cyclophyllidean species. Corresponding tRNA preferences were defined according to the relative abundance of functional tRNA genes decoding each synonymous codon. The magnitude of lineage-associated codon and tRNA divergence was quantified using ΔC and ΔT values, respectively. Based on the consistency of codon and tRNA preference patterns across the GW_CDS, SCO_CDS, and GP_CDS datasets, representative codon families were assigned to strong concordance, moderate concordance, moderate discordance, or strong discordance categories.

## 5. Conclusions

The cestode species examined from the Diphyllobothriidea and Cyclophyllidea exhibited contrasting C-ending and T-ending synonymous codon preferences, respectively, despite relatively conserved amino-acid compositions. These lineage-associated patterns persisted across different CDS datasets and could not be explained solely by nucleotide composition, suggesting contributions from additional evolutionary constraints. Comparative analyses also revealed substantial differences in genomic tRNA repertoires, particularly their reduction and restructuring in cyclophyllidean species. However, the relationships between codon preferences and corresponding tRNA gene abundances varied considerably among synonymous codon families, indicating that their evolutionary associations are not uniformly coordinated. Together, these findings provide a comparative framework for investigating the evolutionary relationships among synonymous codon usage, genomic architecture, and translational systems in parasitic cestodes.

## Figures and Tables

**Table 1:** Number, length, GC content, and effective number of codons (ENC) of CDS datasets across four cestode species.

Dataset and Species	Number	Length in bp (Mean ± SD)	% GC (Mean ± SD)	% GC_1_ (Mean ± SD)	% GC_2_ (Mean ± SD)	% GC_12_ (Mean ± SD)	% GC_3_ (Mean ± SD)	ENC_obs_ (Mean ± SD [Min–Max])	ENC_exp_ − ENC_obs_ ^a^ (Mean ± SD)
GW_CDS									
*Schistocephalus solidus* (43.8%) ^b^	17,659	1079.14 ± 1051.71	51.80 ± 4.37	56.06 ± 5.93	44.49 ± 7.28	50.28 ± 5.07	54.86 ± 6.91	55.83 ± 2.58 (26.57–61.35)	4.08 ± 2.15/3.20 ± 2.00
*Spirometra erinaceieuropaei* (46.0%)	29,318	617.22 ± 347.42	53.37 ± 4.56	57.18 ± 6.21	45.33 ± 7.62	51.25 ± 5.41	57.59 ± 6.88	54.91 ± 2.29 (28.41–60.95)	4.21 ± 2.25/3.45 ± 1.99
*Echinococcus granulosus* (42.1%)	9356	1639.37 ± 1733.57	49.67 ± 3.95	54.25 ± 5.07	43.34 ± 5.89	48.80 ± 4.17	51.42 ± 7.24	56.53 ± 2.56 (38.14–61.38)	4.07 ± 1.82/2.85 ± 1.95
*Taenia solium* (42.3%)	10,796	1385.62 ± 1349.48	49.67 ± 3.70	54.45 ± 4.80	43.19 ± 5.90	48.82 ± 4.18	51.37 ± 6.13	56.54 ± 2.00 (42.16–61.28)	4.36 ± 1.63/3.10 ± 1.76
SCO_CDS									
*Schistocephalus solidus*	1472	1255.70 ± 1045.54	49.69 ± 3.85	54.95 ± 4.70	42.35 ± 5.89	48.65 ± 4.13	51.77 ± 5.99	56.69 ± 1.80 (42.42–60.58)	4.26 ± 1.51/2.96 ± 1.55
*Spirometra erinaceieuropaei*	1325	685.57 ± 381.71	50.53 ± 4.41	55.22 ± 5.39	42.32 ± 6.56	48.77 ± 4.79	54.05 ± 6.54	55.68 ± 1.79 (47.17–60.38)	4.71 ± 1.86/3.58 ± 1.67
*Echinococcus granulosus*	1368	1527.74 ± 1156.63	48.86 ± 3.45	54.22 ± 4.33	42.00 ± 5.35	48.11 ± 3.78	50.37 ± 5.92	56.92 ± 1.82 (46.80–60.78)	4.02 ± 1.48/2.81 ± 1.66
*Taenia solium*	1366	1461.65 ± 1112.94	49.03 ± 3.25	54.49 ± 4.24	42.01 ± 5.37	48.25 ± 3.77	50.60 ± 5.53	56.83 ± 1.78 (48.49–60.72)	4.16 ± 1.46/2.98 ± 1.69
GP_CDS									
*Schistocephalus solidus*	43	1014.98 ± 403.64	48.10 ± 2.61	55.12 ± 3.91	39.17 ± 4.06	47.14 ± 2.88	50.03 ± 5.03	56.05 ± 1.88 (50.73–58.67)	5.28 ± 1.46/3.90 ± 1.75
*Spirometra erinaceieuropaei*	43	1022.09 ± 396.99	49.45 ± 2.36	55.51 ± 4.02	39.21 ± 3.81	47.36 ± 2.99	53.63 ± 5.15	55.76 ± 2.14 (48.93–59.32)	5.27 ± 1.41/3.93 ± 1.62
*Echinococcus granulosus*	43	1026.84 ± 387.90	47.96 ± 2.84	55.27 ± 3.79	39.44 ± 3.91	47.35 ± 3.01	49.18 ± 5.97	54.75 ± 2.49 (46.89–59.24)	5.22 ± 1.58/4.96 ± 2.46
*Taenia solium*	43	1019.09 ± 396.11	48.08 ± 2.80	55.43 ± 3.87	39.23 ± 3.82	47.33 ± 2.99	49.57 ± 5.47	54.54 ± 2.44 (48.49–59.02)	5.25 ± 1.53/5.29 ± 2.56

^a^ ENC_obs_, observed ENC; ENC_exp_, expected ENC. Values before and after the slash are DAMBE ENC_exp_− ENC_obs_ and Wright’s ENC_exp_ − ENC_obs_, respectively. ^b^ GC content in whole genomic sequences of each species.

**Table 2:** Concordance and discordance between lineage-associated codon preferences and corresponding tRNA gene repertoires across representative two-fold degenerate codon families.

AA Family	Pair Type ^a^	CDS Dataset	Codon Preference (Di/Cy) ^b^	ΔC ^c^	tRNA Preference (Di/Cy) ^d^	ΔT ^e^	Classification ^f^	Comments
Asn	C/T	GW	AAC/AAT	0.077	AAT/AAC	0.558	Strong discordance	Consistent codon-tRNA opposition across all datasets with large ΔT.
		SCO	AAC/AAT	0.053	AAT/AAC	0.558		
		GP	AAC/AAT	0.061	AAT/AAC	0.558		
Asp	C/T	GW	GAC/GAT	0.079	GAC/GAC	0.051	Moderate discordance	Partial codon-tRNA opposition with weakened discordance in GP.
		SCO	GAC/GAT	0.061	GAC/GAC	0.051		
		GP	GAT/GAT	0.063	GAC/GAC	0.051		
Cys	C/T	GW	TGC/TGC	0.029	TGC/TGC	0.257	Strong concordance	Consistent codon-tRNA concordance across all datasets.
		SCO	TGC/TGC	0.007	TGC/TGC	0.257		
		GP	TGC/TGC	0.007	TGC/TGC	0.257		
His	C/T	GW	CAC/CAC	0.024	CAC/CAC	0.059	Moderate concordance	Partial codon-tRNA concordance with weakened codon divergence in SCO and GP.
		SCO	CAC/CAT	0.003	CAC/CAC	0.059		
		GP	CAT/CAT	0.007	CAC/CAC	0.059		
Phe	C/T	GW	TTC/TTC	0.032	TTT/TTC	0.882	Moderate discordance	Large tRNA divergence despite limited codon divergence.
		SCO	TTC/TTC	0.000	TTT/TTC	0.882		
		GP	TTC/TTC	0.011	TTT/TTC	0.882		
Tyr	C/T	GW	TAC/TAC	0.037	TAC/TAC	0.064	Moderate concordance	General concordance with minimal codon divergence in GP.
		SCO	TAC/TAC	0.016	TAC/TAC	0.064		
		GP	TAT/TAT	0.003	TAC/TAC	0.064		
Gln	A/G	GW	CAG/CAA	0.062	CAA/CAG	0.182	Strong discordance	Consistent codon-tRNA opposition across all datasets.
		SCO	CAA/CAA	0.039	CAA/CAG	0.182		
		GP	CAA/CAA	0.014	CAA/CAG	0.182		
Glu	A/G	GW	GAA/GAA	0.010	GAA/GAA	0.282	Strong concordance	Consistent codon-tRNA concordance across all datasets with substantial ΔT.
		SCO	GAA/GAA	0.013	GAA/GAA	0.282		
		GP	GAA/GAA	0.018	GAA/GAA	0.282		
Lys	A/G	GW	AAA/AAA	0.020	AAA/AAG	0.369	Moderate discordance	Consistent tRNA opposition despite limited codon divergence.
		SCO	AAA/AAA	0.006	AAA/AAG	0.369		
		GP	AAA/AAG	0.021	AAA/AAG	0.369		

^a^ Pair type indicates whether synonymous codons differ at C/T or A/G at the third codon position. ^b^ Codons exhibiting higher mean observed frequencies in the diphyllobothriidean (Di; *Schistocephalus solidus* and *Spirometra erinaceieuropaei*) and cyclophyllidean (Cy; *Echinococcus granulosus* and *Taenia solium*) lineages. ^c^ Absolute difference between lineage-specific codon preference values (ΔC). ^d^ Codons decoded by tRNA anticodons with the higher functional tRNA gene counts in the corresponding lineage. ^e^ Absolute difference between lineage-specific tRNA preference values (ΔT). ^f^ Codon–tRNA relationships were assessed qualitatively by comparing synonymous codon preferences with the relative genomic counts of their corresponding functional tRNA genes. Concordance and discordance indicate agreement and opposition between these preferences, respectively. Strong and moderate relationships were distinguished descriptively according to the apparent consistency of these preferences across the examined species and three CDS datasets, rather than by fixed numerical thresholds.

## Data Availability

The data used to support the findings of this study are available from the corresponding author (Bae, Y.-A.) upon request.

## Supplementary Materials

The following supporting information can be downloaded at: https://www.mdpi.com/article/10.3390/ijms27209282/s1.

## Abbreviations

The following abbreviations are used in this manuscript:

ANCOVA	Analysis of covariance
CDS	Coding DNA sequence
CUB	Codon usage bias
ENC	Effective number of codons
FDR	False discovery rate
FOP	Frequency of optimal codons
LOESS	Locally weighted scatterplot smoothing
PR2	Parity Rule 2
RSCU	Relative synonymous codon usage
SCO	Single-copy ortholog
