# Supplementary material for: Lineage-Associated Dichotomous Patterns in Codon Usage of Parasitic Cestodes
Source: Int J Mol Sci. 2026 Oct 19;27(20):9282. doi: 10.3390/ijms27209282 (PMC13705341; doi:10.3390/ijms27209282)
Supplement: Supplementary file 1 [file ijms-27-09282-s001.zip › Kim et al-Cesotde CUB_Suppl figures S1 - S9.pdf]

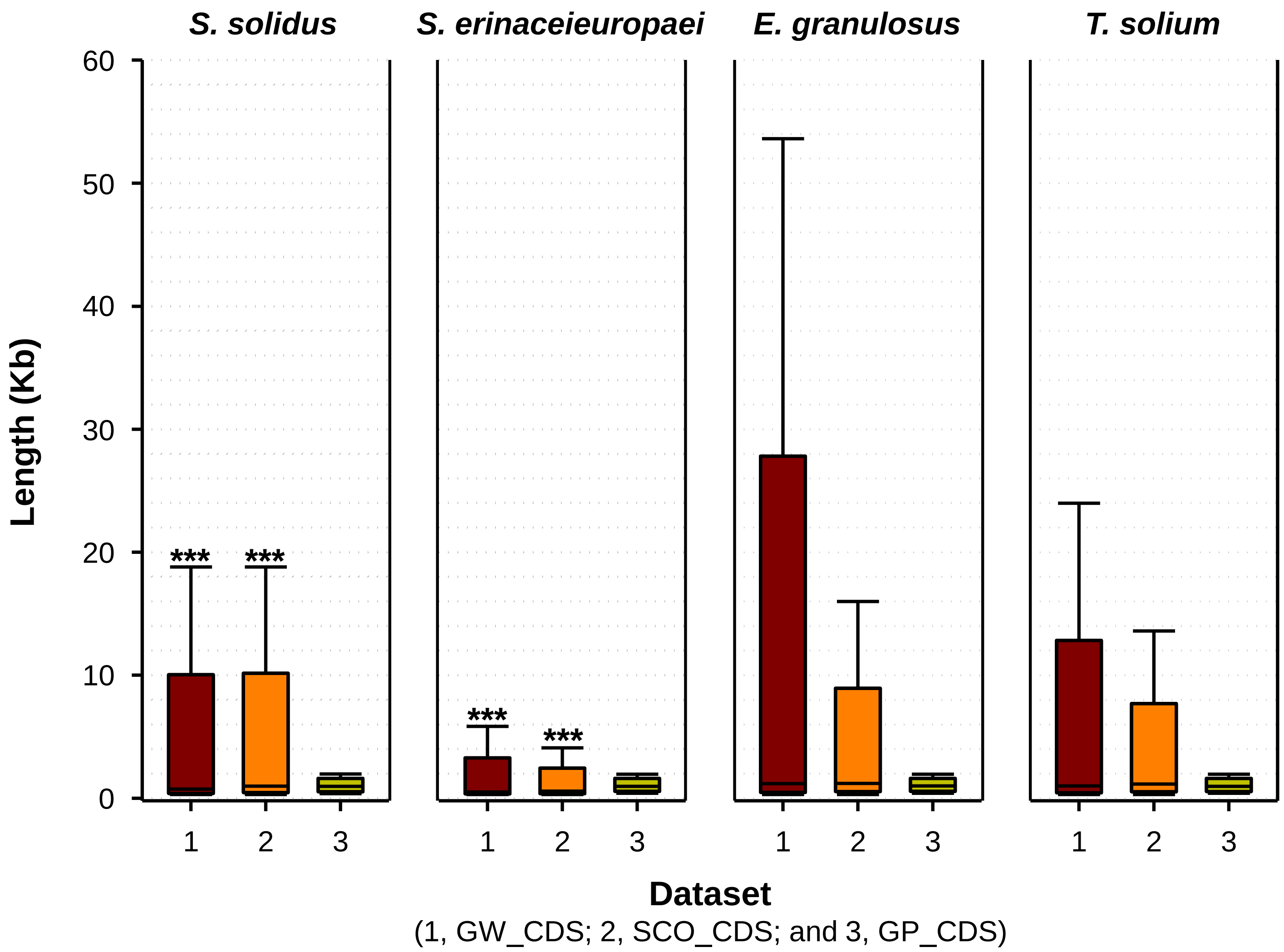

Supplementary Figure S1. Length distributions of coding sequences across cestode CDS datasets. Box plots show CDS length distributions in the GW_CDS, SCO_CDS, and GP_CDS datasets for *Schistocephalus solidus*, *Spirometra erinaceieuropaei*, *Echinococcus granulosus*, and *Taenia solium*. The horizontal line within each box indicates the median CDS length. CDS lengths differed significantly among species in the GW_CDS and SCO_CDS datasets (Kruskal–Wallis tests, $P < 0.05$), whereas no significant interspecific difference was detected in the GP_CDS dataset (Kruskal–Wallis test, $P = 0.378$). Pairwise differences involving *S. erinaceieuropaei* were assessed using one-sided Mann–Whitney U tests with Holm correction, testing whether its CDSs were shorter than those of the other species, whereas two-sided Mann–Whitney U tests were used for *S. solidus* because the differences were not consistently directional. Asterisks indicate significant pairwise differences (***$P < 0.001$).

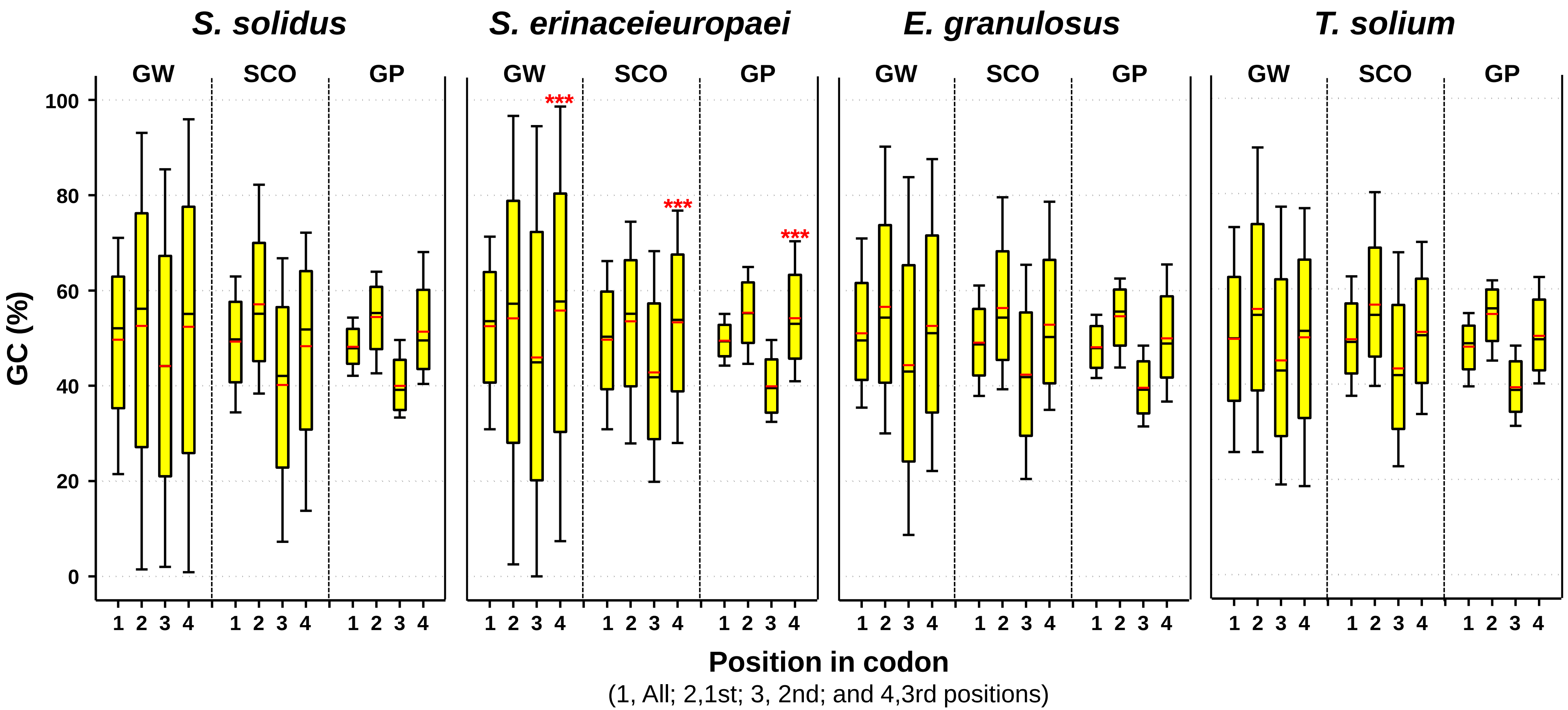

Supplementary Figure S2. GC composition across codon positions in cestode CDS datasets. Box plots show GC contents at all codon positions combined and at the first, second, and third codon positions separately across the GW_CDS, SCO_CDS, and GP_CDS datasets for *Schistocephalus solidus*, *Spirometra erinaceieuropaei*, *Echinococcus granulosus*, and *Taenia solium*. Black and red horizontal lines within the boxes indicate median and mean values, respectively. GC_3 differed significantly among species in all three CDS datasets (one-way ANOVA or Kruskal–Wallis tests, ***$P < 0.001$), with *S. erinaceieuropaei* consistently showing the highest values.

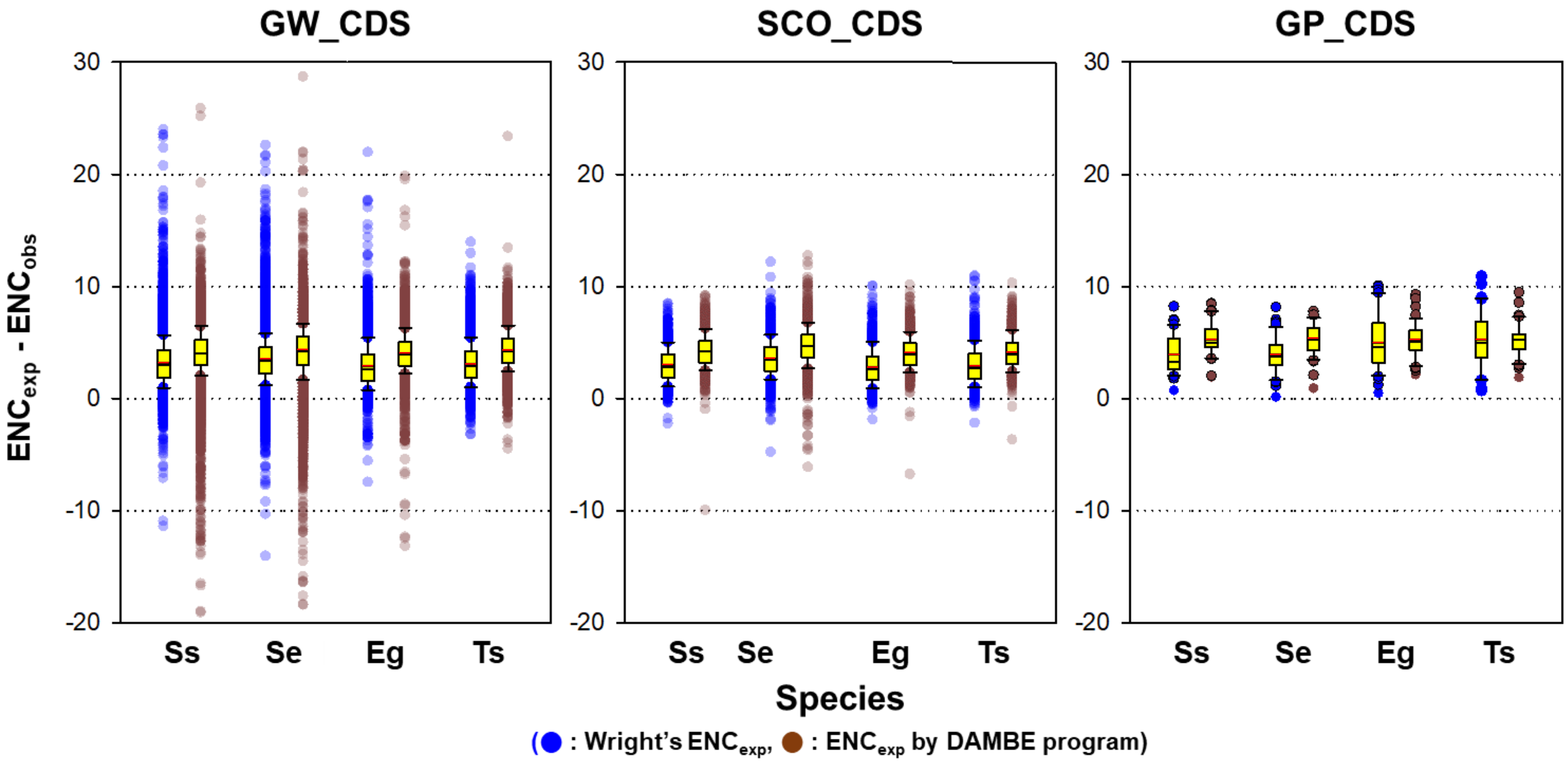

Supplementary Figure S3. Distribution of ENC deviations. Deviations between observed and expected ENC values (ΔENC, calculated as $ENC_{exp} - ENC_{obs}$) are shown for the GW_CDS, SCO_CDS, and GP_CDS datasets of *Schistocephalus solidus* (Ss), *Spirometra erinaceieuropaei* (Se), *Echinococcus granulosus* (Eg), and *Taenia solium* (Ts). Blue and brown circles represent ΔENC values calculated using Wright- and DAMBE-derived expected ENC values, respectively, and box plots summarize their distributions. Black and red horizontal lines within the boxes indicate median and mean values, respectively.

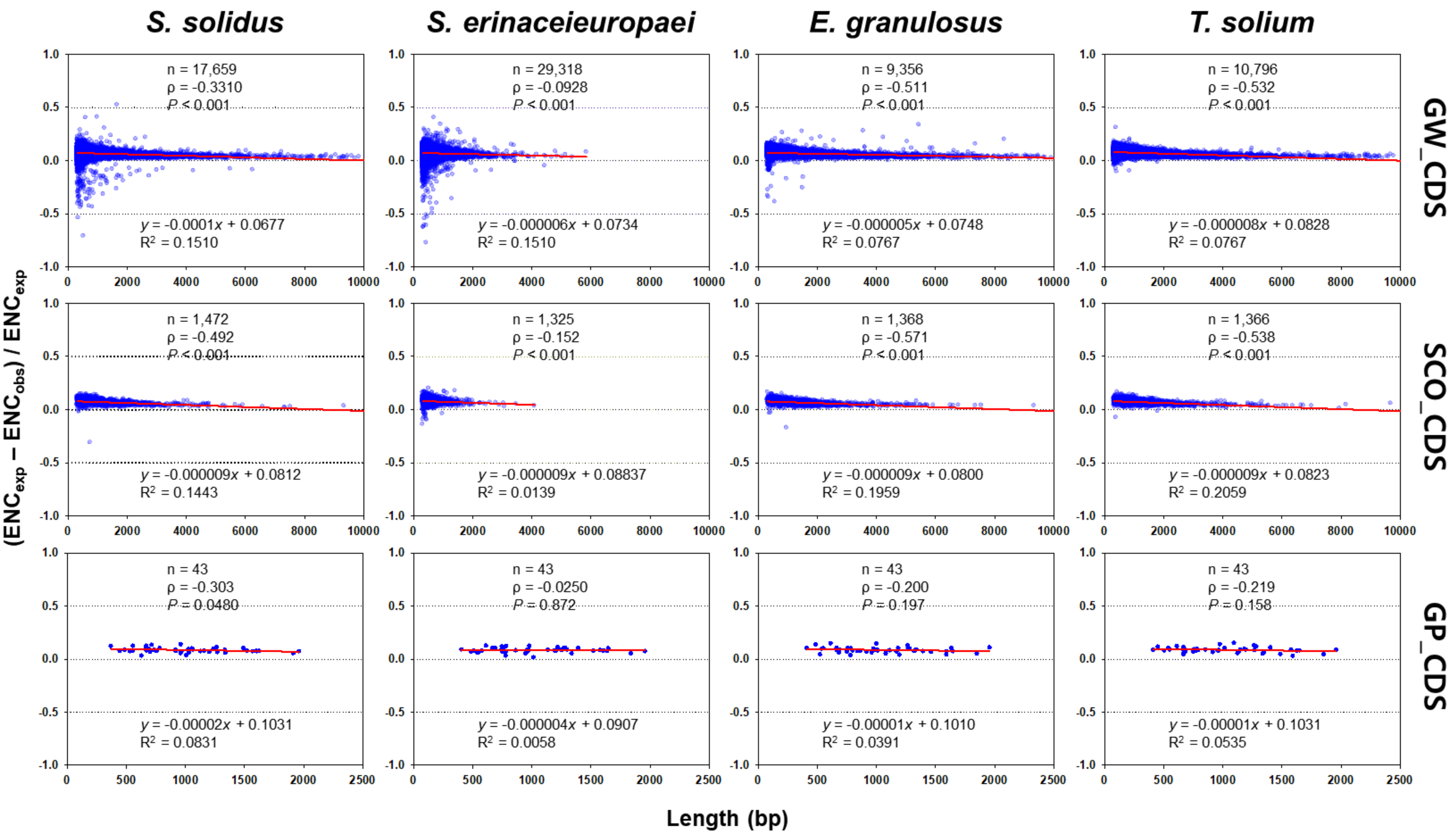

Supplementary Figure S4. Relationships between CDS length and relative ENC deviation. Scatter plots show the relationships between CDS length and relative ENC deviation, calculated as $(ENC_{exp} - ENC_{obs})/ENC_{exp}$, in the GW_CDS, SCO_CDS, and GP_CDS datasets, with ENC_{exp} calculated using DAMBE. Spearman's correlation coefficients (ϱ) and associated *P* values are shown together with sample sizes (n) in each panel. Red solid lines represent linear regression fits, with the corresponding regression equations and R^2 values shown in each panel.

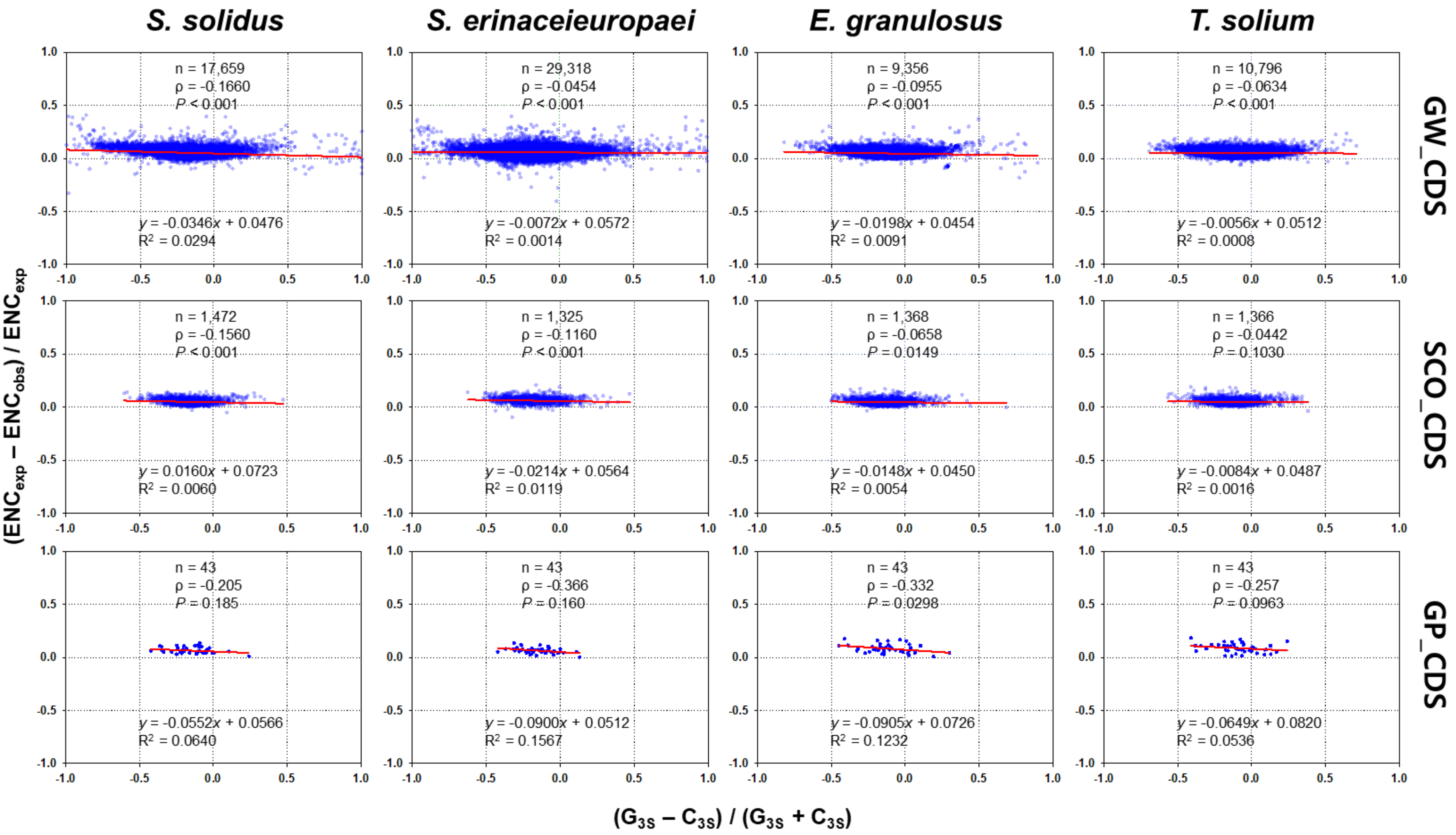

Supplementary Figure S5. Relationships between GC skew and relative ENC deviation. Scatter plots show the relationships between GC skew, calculated as $(G_{3S} - C_{3S})/(G_{3S} + C_{3S})$, and relative ENC deviation, calculated as $(ENC_{exp} - ENC_{obs})/ENC_{exp}$, in the GW_CDS, SCO_CDS, and GP_CDS datasets, with ENC_{exp} calculated using Wright's formula. Spearman's correlation coefficients (ρ) and associated P values are shown together with sample sizes (n) in each panel. Red solid lines represent linear regression fits, with the corresponding regression equations and R^2 values shown in each panel.

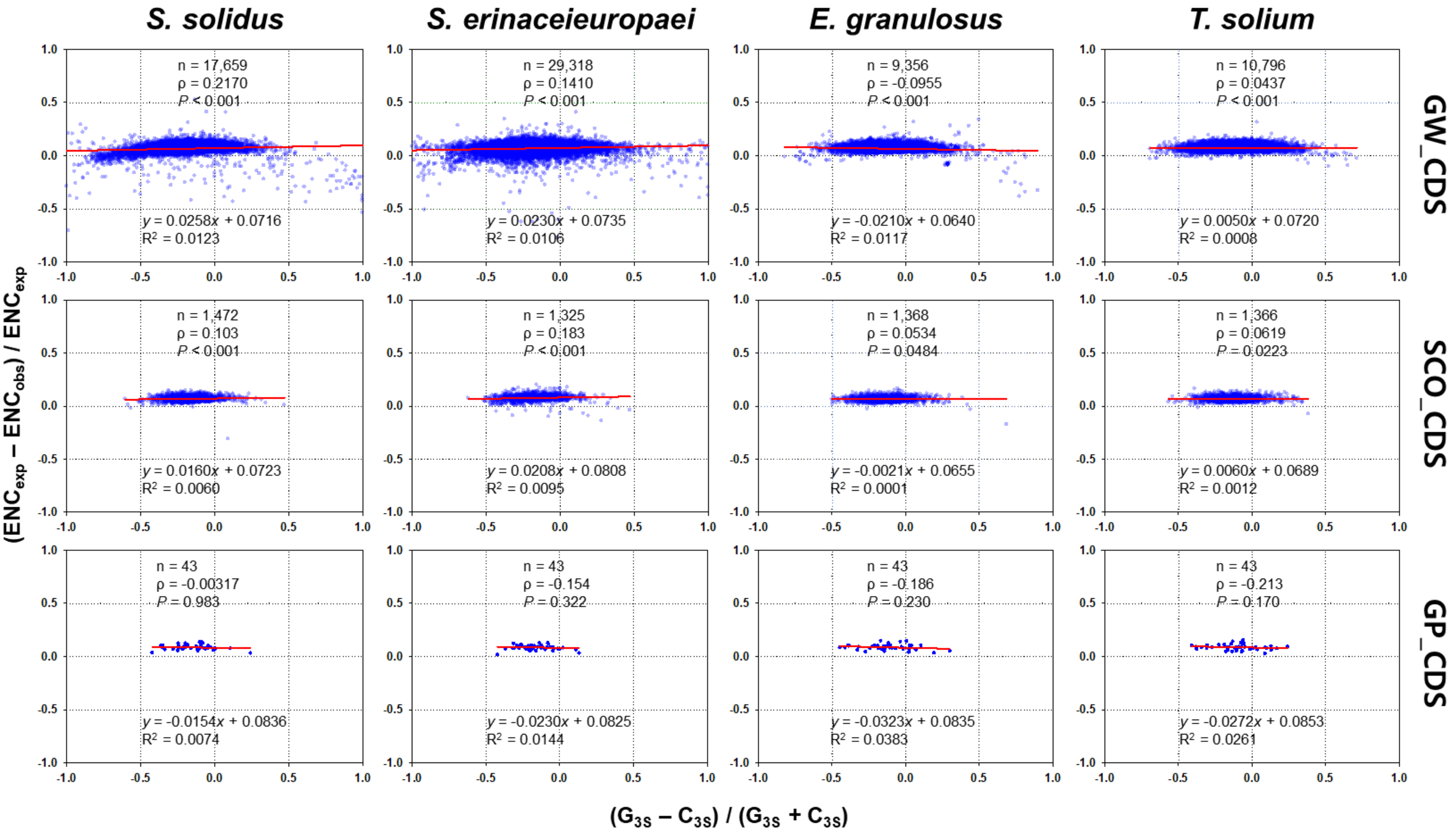

Supplementary Figure S6. Relationships between GC skew and relative ENC deviation. Scatter plots show the relationships between GC skew, calculated as $(G_{3S} - C_{3S})/(G_{3S} + C_{3S})$, and relative ENC deviation, calculated as $(ENC_{exp} - ENC_{obs})/ENC_{exp}$, in the GW_CDS, SCO_CDS, and GP_CDS datasets, with ENC_{exp} calculated using DAMBE. Spearman's correlation coefficients (ρ) and associated *P* values are shown together with sample sizes (n) in each panel. Red solid lines represent linear regression fits, with the corresponding regression equations and R^2 values shown in each panel.

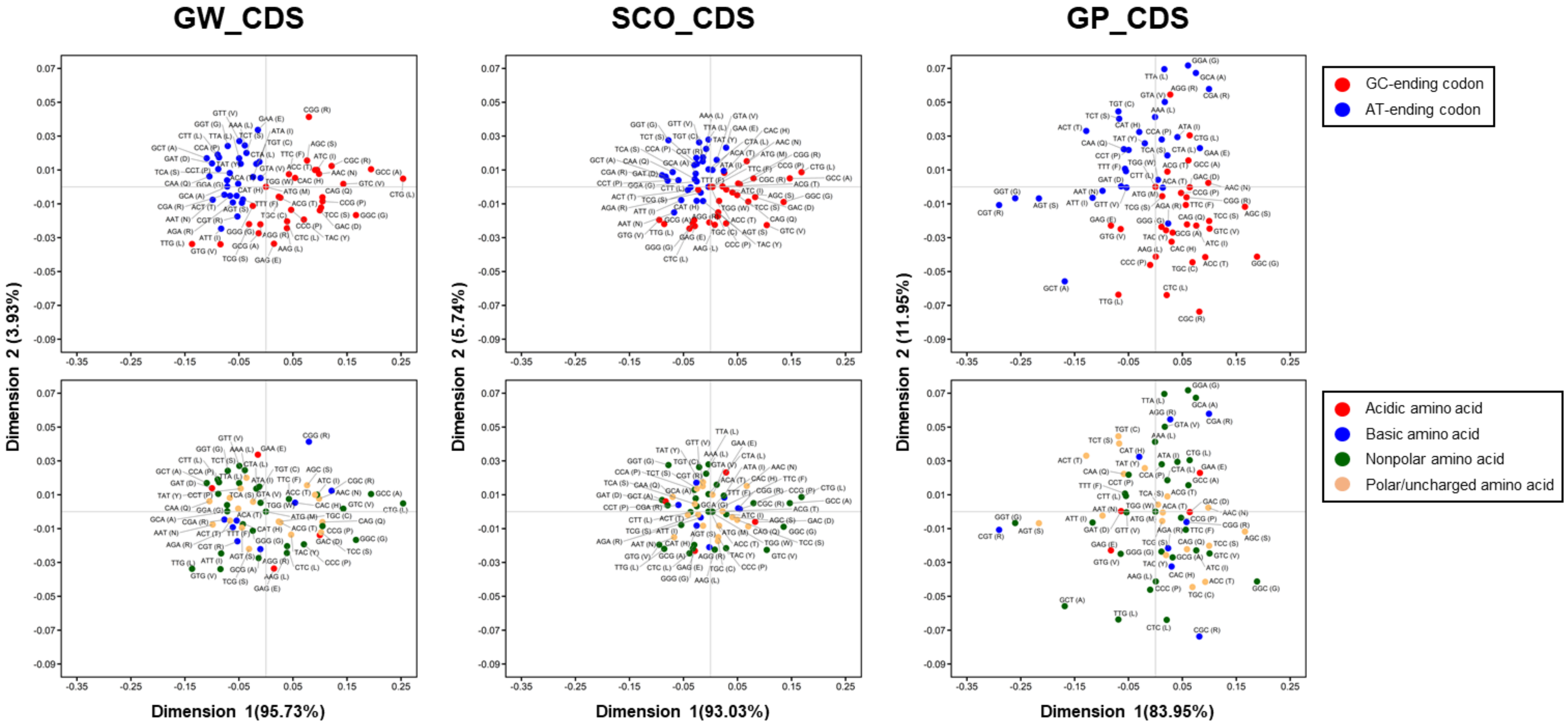

Supplementary Figure S7. Distribution of synonymous codons on correspondence analysis axes. Correspondence analysis plots show the distributions of synonymous codons in the GW_CDS, SCO_CDS, and GP_CDS datasets. GC-ending and AT-ending codons are distinguished in the upper panels. In the lower panels, codons are distinguished according to the physicochemical properties of their corresponding amino acids as acidic, basic, nonpolar, or polar/uncharged.

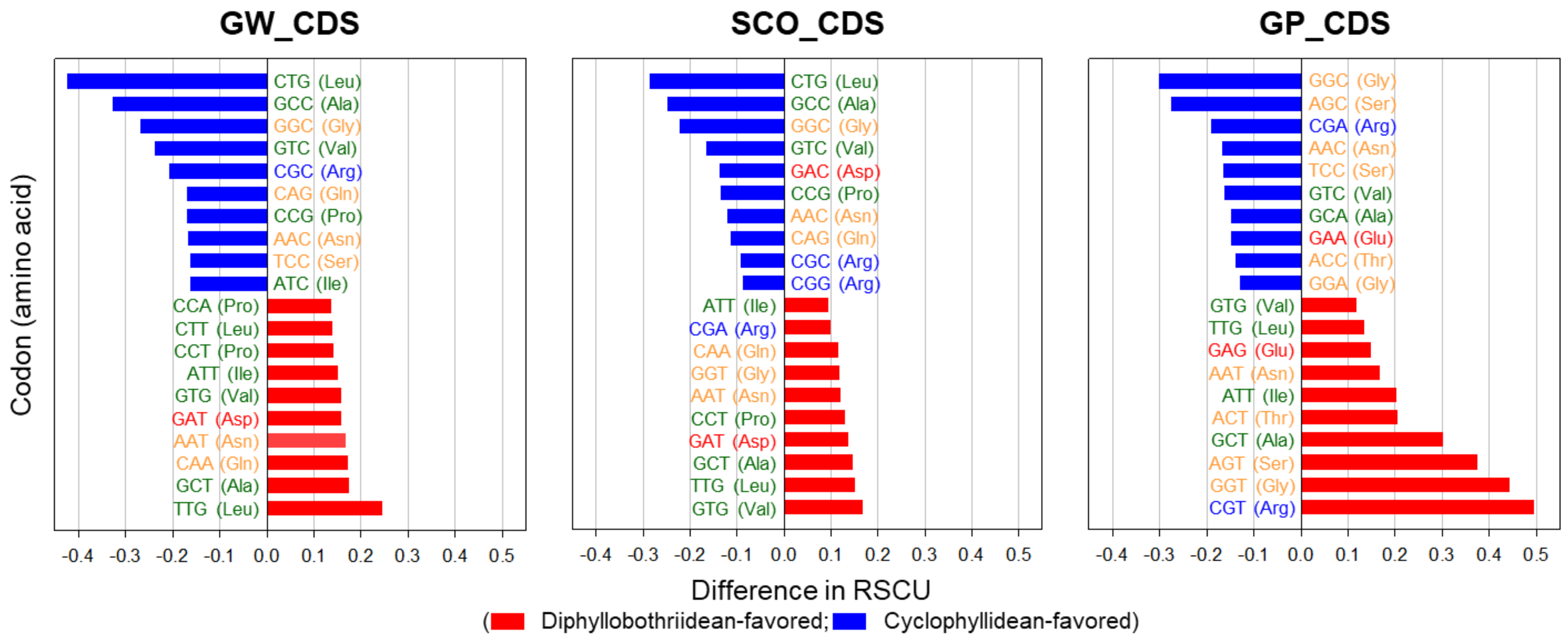

Supplementary Figure S8. Differential relative synonymous codon usage (RSCU) patterns between diphyllobothriidean and cyclophyllidean lineages. Bar plots show differences in RSCU between diphyllobothriidean and cyclophyllidean lineages in the GW_CDS, SCO_CDS, and GP_CDS datasets. Positive (red) and negative (blue) values indicate codons favored in diphyllobothriideans and cyclophyllideans, respectively. Codon labels are colored according to the physicochemical properties of their corresponding amino acids: green, nonpolar; orange, polar/uncharged; red, acidic; and blue, basic.

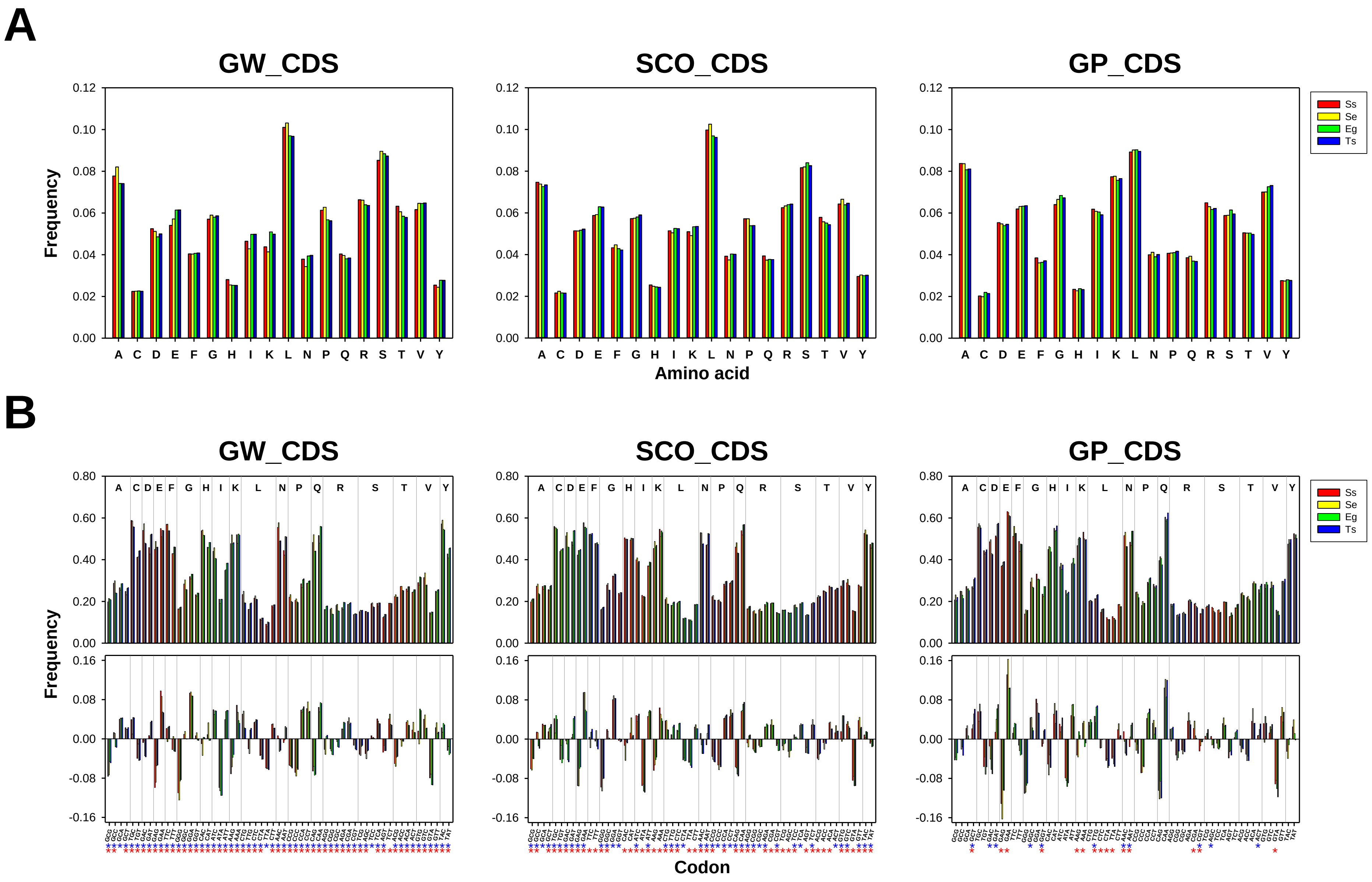

Supplementary Figure S9. Amino acid and synonymous codon frequency profiles across cestode CDS datasets. (**A**) Mean per-gene amino acid frequencies in the GW_CDS, SCO_CDS, and GP_CDS datasets. (**B**) Mean per-gene synonymous codon frequencies (upper panels) and GC_{3S}-corrected residual codon frequencies (lower panels) in the corresponding datasets. Values are shown for *Schistocephalus solidus* (Ss), *Spirometra erinaceieuropaei* (Se), *Echinococcus granulosus* (Eg), and *Taenia solium* (Ts). Blue and red asterisks indicate codons exhibiting significant lineage-associated differences in synonymous codon and GC_{3S}-corrected residual codon frequencies, respectively, after Wilcoxon rank-sum tests with Benjamini–Hochberg false discovery rate (FDR) correction (FDR-adjusted $P < 0.05$).
